# Haplotype Loci Under Selection in Canadian Durum Wheat Germplasm Over 60 Years of Breeding: Association With Grain Yield, Quality Traits, Protein Loss, and Plant Height

**DOI:** 10.3389/fpls.2018.01589

**Published:** 2018-11-05

**Authors:** Amidou N’Diaye, Jemanesh K. Haile, Kirby T. Nilsen, Sean Walkowiak, Yuefeng Ruan, Asheesh K. Singh, Fran R. Clarke, John M. Clarke, Curtis J. Pozniak

**Affiliations:** ^1^Department of Plant Sciences, Crop Development Centre, University of Saskatchewan, Saskatoon, SK, Canada; ^2^Agriculture and Agri-Food Canada, Swift Current Research and Development Centre, Swift Current, SK, Canada; ^3^Department of Agronomy, Iowa State University, Ames, IA, United States

**Keywords:** haplotype, loci under selection, durum wheat, quality traits, grain yield, protein loss, plant height

## Abstract

Durum wheat was introduced in the southern prairies of western Canada in the late nineteenth century. Breeding efforts have mainly focused on improving quality traits to meet the pasta industry demands. For this study, 192 durum wheat lines were genotyped using the Illumina 90K Infinium iSelect assay, and resulted in a total of 14,324 polymorphic SNPs. Genetic diversity changed over time, declining during the first 20 years of breeding in Canada, then increased in the late 1980s and early 1990s. We scanned the genome for signatures of selection, using the total variance Fst-based outlier detection method (Lositan), the hierarchical island model (Arlequin) and the Bayesian genome scan method (BayeScan). A total of 407 outliers were identified and clustered into 84 LD-based haplotype loci, spanning all 14 chromosomes of the durum wheat genome. The association analysis detected 54 haplotype loci, of which 39% contained markers with a complete reversal of allelic state. This tendency to fixation of favorable alleles corroborates the success of the Canadian durum wheat breeding programs over time. Twenty-one haplotype loci were associated with multiple traits. In particular, *hap_4B_1* explained 20.6, 17.9 and 16.6% of the phenotypic variance of pigment loss, pasta b^∗^ and dough extensibility, respectively. The locus *hap_2B_9* explained 15.9 and 17.8% of the variation of protein content and protein loss, respectively. All these pleiotropic haplotype loci offer breeders the unique opportunity for further improving multiple traits, facilitating marker-assisted selection in durum wheat, and could help in identifying genes as functional annotations of the wheat genome become available.

## Introduction

Durum wheat (*Triticum turgidum* L. ssp. *durum* Desf. Husn., 2n = 4x = 28; genome AABB) is an important crop in Canada, grown on an average of approximately 2 million hectares and comprising about 25% of total wheat area (Canada, 2018). Nearly all of Canada’s wheat is produced in the western prairie provinces of Saskatchewan, Alberta and Manitoba, with a relatively small area in British Columbia and eastern Canada (McCallum and DePauw, 2008). Durum wheat was introduced into western Canada in the late nineteenth century (see Dexter, 2008 for a detailed history of Canadian durum wheat breeding) and planned hybridization and targeted selection started in 1928 (Clarke J.M. et al., 2010). However, the first variety developed in Canada, Stewart 63, was not released until 1963 (Dexter, 2008).

The improvement of quality traits, such as yellow pigment and gluten strength, was a major focus for durum breeding to satisfy the requirements of the global pasta industry. Canadian durum wheat is classified into four Canada Western Amber Durum (CWAD) wheat milling grades defined by the Canadian Grain Commission (Dexter and Edwards, 1998). Only varieties that meet a set of requirements for a grade are registered. Specifications for new varieties continue to evolve in response to the feedback of CWAD customers (Dexter, 2008). Durum breeding in Canada has made steady genetic progress to improve yield and agronomic traits. This was done concomitantly with improvements in end-use quality attributes such as grain protein concentration, yellow pigment concentration and gluten strength, while improving or maintaining resistance to disease (Clarke J.M. et al., 2010). Grain protein concentration and gluten strength are crucial factors in pasta manufacturing and cooking quality (Feillet and Dexter, 1996). These and other quality trait targets have indirectly driven durum wheat breeders to design hybridization programs within narrow limits, using a similar set of standard cultivars as donors of these quality traits. In particular, high grain protein concentration is a requirement for durum cultivar registration in Canada and this likely limited grain yield gain (Clarke J.M. et al., 2010) due to the generally negative relationship between grain yield and protein concentration (Clarke et al., 2009).

When it is necessary to bring new diversity for particular traits into the breeding programs, it is imperative to return the elite materials to a new state of equilibrium as quickly as possible. Efficient means to identify both core essential adaptation and quality traits in addition to new traits being introgressed can facilitate this process. Starting in the late 1990s, molecular markers became an important tool for Canadian wheat breeding programs. However, the lack of tightly linked diagnostic markers, QTL × environmental interactions and prevalence of QTL background effects have limited the application of marker assisted breeding for some traits (Randhawa et al., 2013). Advances in high-throughput genotyping platforms at a low cost have now made it possible to consider using empirical LD patterns to conduct genome-wide scans to link markers with phenotypes of interest. Additionally, the recent availability of high quality genome assemblies for tetraploid wheat (Avni et al., 2017) could be a valuable source to facilitate the identification of loci and genes of interest.

Association mapping (AM) is increasingly being adopted as a complementary method to bi-parental linkage mapping to identify genotype–phenotype associations. Several AM studies have been conducted to dissect the genetic basis of durum grain yield (Maccaferri et al., 2011; Mengistu et al., 2016; Kidane et al., 2017; Sukumaran et al., 2018), grain and semolina quality traits (Fiedler et al., 2017), semolina and pasta color (N’Diaye et al., 2017), agronomic and morphological traits (Hu et al., 2015) and grain cadmium concentration and yellow color loss during pasta manufacturing (Pozniak et al., 2012) in durum wheat. Recently, the haplotype-based AM approach was suggested as an efficient method for investigating the genetic basis of traits of interest in durum wheat by detecting more loci (N’Diaye et al., 2017), capturing epistatic interactions and reducing type I error rate (Morris and Kaplan, 2002; Zhao et al., 2007; Hamblin and Jannink, 2011). All of these genome-wide association studies in durum wheat populations were performed using the whole set of polymorphic SNPs, but not loci under selection over the course of durum wheat breeding. However, the detection of selection signatures is gaining ground in modern population genetics (Fariello et al., 2013). The growing availability of large-scale genotyping data has facilitated the identification of regions targeted by natural and/or artificial selection in wild and domesticated populations of plants, animals and humans (Nielsen et al., 2007). The search for molecular signatures aims to uncover the evolutionary past of species, understand their functional or adaptive importance, and detect associations between these genomic regions and traits of interest (López et al., 2015). It has become an efficient approach in biomedical sciences to identify genes related to disease resistance (Tishkoff et al., 2001; Barreiro et al., 2008; Albrechtsen et al., 2010; Fumagalli et al., 2010; Cagliani et al., 2011), adaptation to climate (Lao et al., 2007; Sturm, 2009; Rees and Harding, 2012), or altitude (Bigham et al., 2010; Simonson et al., 2010). In livestock species, where artificial selection has been carried out by humans since domestication, it contributes to mapping traits of commercial interest (Guan et al., 2016; Liu et al., 2016; Taye et al., 2017).

Therefore, the main objectives of this study were to scan the genome of elite Canadian durum wheat lines tested in the pre-registration trial for selection footprints and relate these genomic regions to phenotypes. The availability of such loci targeted by selection would be a valuable resource for developing markers and/or investigating gene candidates controlling the traits of interest we analyzed, in particular grain yield, protein loss and pasta quality traits.

## Materials and Methods

### Plant Materials and Phenotypic Data Analyses

One hundred and ninety-two durum wheat lines, including the 169 lines from our previous study (N’Diaye et al., 2017) from the official Canadian durum cultivar registration trial (Durum wheat Cooperative Test) were used for this project (Supplementary Table S1). Phenotypic data and trials were described in previous reports (Clarke J.M. et al., 2010; Pozniak et al., 2012; Haile et al., 2018). Briefly, the trials were run under the auspices of science/industry groups responsible for recommending cultivars for registration by the Canadian Food Inspection Agency and grown each year at 10–12 locations in western Canada and one in the United States. Candidate lines were tested for 1–3 years, those that did not meet the merit requirements being withdrawn, with 3 years of data required for registration of cultivars. Each trial included four or five check cultivars. The checks AC Avonlea, AC Morse, AC Navigator and Strongfield were in the trials for the years since 1999, and Commander since 2001. Trials were arranged in lattice designs with four replications. For the end-use quality traits, we evaluated gluten index described by Haile et al. (2018), semolina pigment, semolina b^∗^, pasta b^∗^ and pigment loss as previously described (N’Diaye et al., 2017). We also included alveograph measures (Haile et al., 2018): dough tenacity (P), dough extensibilty (L), and deformation energy (W). Protein loss was estimated as the difference between grain protein and semolina protein concentration.

The historical and unbalanced phenotypic data were analyzed using SAS version 9.4 PROC HPMIXED with three models due to the different data structures to calculate best linear unbiased predictions (BLUPs). For grain yield concentration which was measured on composites of locations within years (175 lines), year, location, replication, and genotype, and their interactions were considered random; for grain protein and yellow pigment concentrations (186 lines), years, locations, genotypes, and interactions were random; for gluten strength and color traits measured on yearly composites (170 lines), genotypes and years were random. The analyses included all genotypes tested (up to approximately 300 depending on trait) in the registration trials, not just those used in the present study, to provide a better estimate of random variances and covariances (Clarke F.R. et al., 2010; Pozniak et al., 2012).

### SNP Genotyping and Data Curation

DNA extraction and genotyping with the 90K iSelect assay chip were carried out as reported in our previous paper (N’Diaye et al., 2017). A total of 14,324 polymorphic SNPs were scored and missing calls were imputed using the RF regression procedure (Breiman, 2001) as implemented in the randomForest R package (Liaw and Wiener, 2002; R Development Core Team, 2013). After removing SNPs having MAF < 0.05, a total of 11,323 SNPs were kept for analyses.

Because relatively few semi-dwarf lines in the panel were selected for very high pigment, presenting the possibility of spurious associations, the lines were also genotyped with *Rht-B1b*, an allele known to confer semi-dwarf growth habit in wheat (Ellis et al., 2002). In order to distinguish the association signals from *Rht-B1b*, pairwise LD (*r*2) was performed between all 4B association signals and this gene using MIDAS software (Gaunt et al., 2006).

### Population Structure and Genetic Diversity Analysis

First, duplicate SNPs were removed using an in-house Ruby script, as previously described (N’Diaye et al., 2017). Then, a subset of 4,235 highly polymorphic (0.32 ≤ PIC ≤ 0.45) SNPs were selected for the clustering analysis (Kabbaj et al., 2017). The population structure among the 192 breeding lines was investigated using the discriminant analysis of principal components (DAPC) as implemented in the Adegenet R package (Jombart, 2008; Jombart et al., 2010; Jombart and Ahmed, 2011). The number of clusters (K) for the DAPC was estimated from the lowest value of the Bayesian information criterion (BIC) according to Jombart et al. (2010).

The genetic differentiation between populations derived from the DAPC was assessed by pairwise Fst values, and the variance between and within populations was calculated using the analysis of molecular variance (AMOVA) as implemented in the Arlequin 3.5 software (Excoffier and Lischer, 2010). Significance levels for variance components and Fst statistics were estimated based on 10,000 permutations. To analyze the changes in diversity over time, the breeding lines were assigned to temporal groups (decades) according to the time of entry into the Durum wheat Cooperative Test and the genetic diversity (π) was computed according to Nei and Li (1979), using Arlequin 3.5 software (Excoffier and Lischer, 2010).

### Selection Signatures

The subset of 4,235 highly polymorphic SNPs was used to scan the genome for signatures of selection, using three approaches: the total variance Fst-based outlier detection method implemented in Lositan (Antao et al., 2008), the hierarchical island model with 100,000 simulations using Arlequin (Excoffier and Lischer, 2010) and the Bayesian genome scan approach implemented in BayeScan (Foll and Gaggiotti, 2008). For the latter, we performed 20 pilot runs of 50,000 iterations, followed by 100,000 iterations on a sample size of 5000 and thinning interval of 10.

For Lositan, markers were considered under divergent selection if the Fst values were higher than 99% of the neutral distribution. For Arlequin, loci were considered being under selection if the observed Fst values were higher than expected on the basis of neutral variation, and showing Fst out of the 99% quantile based on coalescent simulations (Beaumont and Nichols, 1996). We identified markers under selection with BayeScan by comparing posterior probabilities and threshold values obtained from the FDR (FDR *q*-values < 0.05). As a result, the Fst cut-off for declaring outliers was 0.20 for Arlequin and Lositan, and 0.15 for BayeScan (Supplementary Figure S1).

### Haplotype Blocks and Association Study

Haplotype blocks were built as previously described (N’Diaye et al., 2017), with little modifications. Only markers that showed strong evidence of directional selection were clustered into haplotype blocks based on the durum wheat consensus map (Maccaferri et al., 2014), using an in-house python script. These haplotype loci were used to perform association studies in order to relate them to the traits of interest (e.g., gluten index, semolina pigment, grain yield, protein content, protein loss, and plant height) that have been scored over years. The haplotype-trait association analyses were carried out using a Mixed Linear Model (MLM) (Yu et al., 2006) with either the Kinship matrix alone (MLM-K) or the Q matrix from the DAPC plus Kinship (MLM-QK) as random effect, using TASSEL software version 3 (Bradbury et al., 2007). The Q-Q (quantile-quantile) plot profiles (Supplementary Figure S2) showed that MLM-K better controlled the *P*-value inflation while MLM-QK led to overcorrections. Therefore, all association analyses were carried out using the MLM-K model. A false discovery rate (FDR) of 5% was applied and haplotype loci with *P*-value ≤ 0.05 were declared significant. The allelic effect of haplotypes was estimated as the difference between the mean value of the lines carrying these haplotypes and the mean value of the entire population for each trait, as previously described (N’Diaye et al., 2017).

## Results

### Genetic Diversity and Population Stratification

**FIGURE 1:**
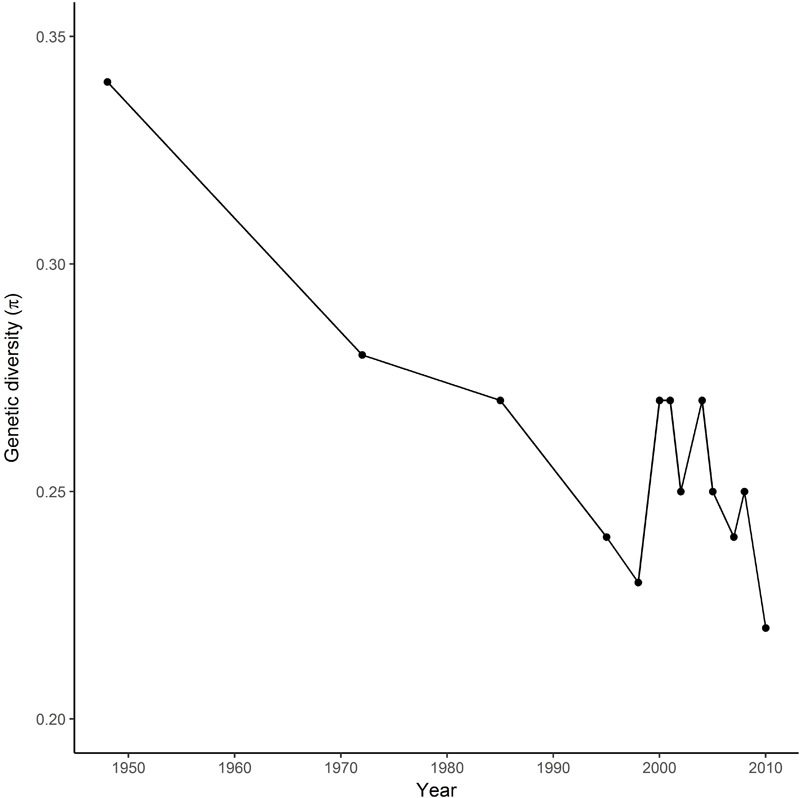
Changes in genetic diversity (π) of the Durum wheat Cooperative Test in Canada from 1950 to 2010.

SNP markers used for the analysis were distributed across all 14 chromosomes of the durum wheat genome. Genetic diversity in the registration trials changed over time, declining during the first 20 years of breeding in Canada when the germplasm shifted from introduced cultivars in the 1940s and 1950s to locally-bred lines in the 1970s (Figure 1). Diversity increased in the late 1980s and early 1990s, then started a decrease in the 2005s.

**FIGURE 2:**
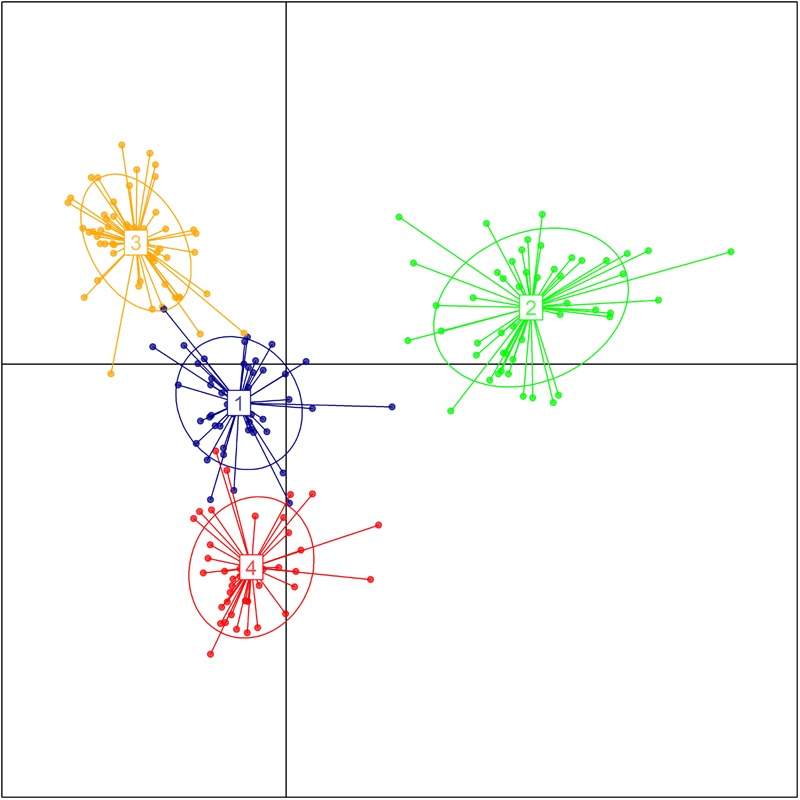
Population structure of the breeding panel as revealed by discriminant analysis of principal components. The axes represent the first two Linear Discriminants. Each color represents a sub-population.

The genetic relationship between the 192 durum lines as revealed by the DAPC is illustrated in Figure 2. Based on the lowest BIC, the lines were clustered into four sub-populations. However, the pairwise Fst analysis showed only moderate (0.05 < Fst ≤ 0.15) genetic differentiation between the four sub-populations (Table 1). The AMOVA revealed that only 9.8% of the genetic variation is found between sub-populations, whereas 85.2% of the genetic variation resides between individuals (Table 2).

**Table 1:** Pairwise Fst between subpopulations, SP1 (*n* = 45), SP2 (*n* = 50), SP3 (*n* = 57), and SP4 (*n* = 40).

Sub-populations	SP1	SP2	SP3
SP2	0.11		
SP3	0.06	0.15	
SP4	0.07	0.10	0.09

**Table 2:** Analysis of molecular variance (AMOVA).

Sources of variation	d. f.	Sum of squares	Variance components	Variation (%)
Populations (P)	3	22,270.31^∗∗∗^	70.30	9.80
Individuals within P	188	129,003.06^∗∗∗^	35.85	5.00
Individuals	192	117,895.00^∗∗∗^	610.85	85.20
Total	383	269,168.37	717.00	

*^∗∗∗^The source of variation was highly significant at P ≤ 0.001.*

### Loci Under Selection

**FIGURE 3:**
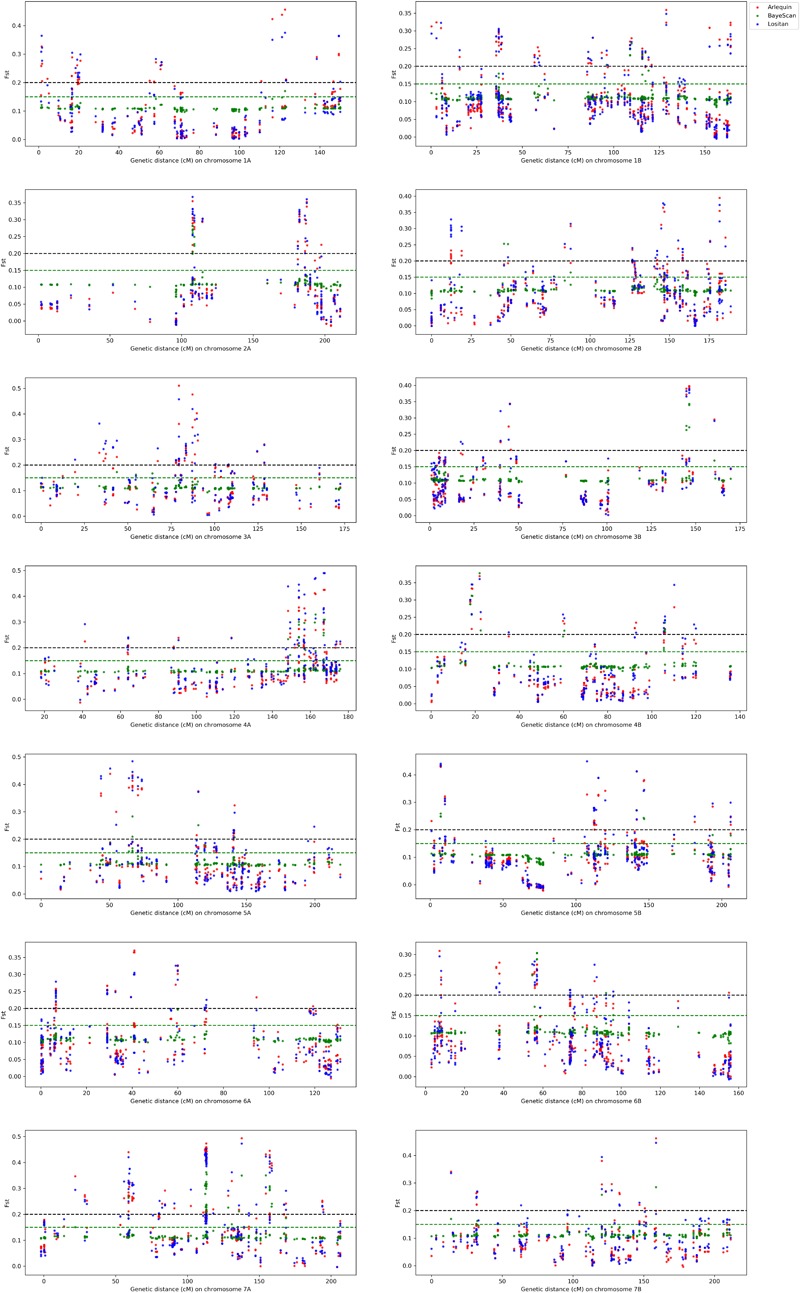
Genome-wide analysis of loci under selection using Arlequin (red), BayeScan (green), and Lositan (blue). The horizontal black dash line indicates the threshold for selection with Arlequin and Lositan while the horizontal green dash line shows the selection threshold for BayeScan.

**FIGURE 4:**
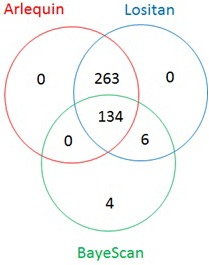
Venn diagram of loci under selection detected by Arlequin, BayeScan, and Lositan.

The Fst distribution and threshold (0.15 for BayeScan, 0.2 for both Arlequin and Lositan) to declare loci being under selection are shown on Supplementary Figure S1. From the total of 4,235 most informative SNPs, 407 appeared to be under selection, spanning all 14 chromosomes of the durum wheat genome (Figure 3). Lositan detected 403 outliers, including all 397 markers from Arlequin (Figure 4). BayeScan identified only 144 outliers, of which 4 SNPs were undetected by Lositan and BayeScan. Twenty-three percent (95/407) of markers under selection showed a complete reversal of allelic state in response to selection over time. However, the time at which the allelic state changed was different depending on the marker. For example, the switch of allele’s frequency occurred in the early 1960s (e.g., BobWhite_c8016_301, BS00009060_51, and BS00087544_51), in the mid-1980s (BS00091561_51, BS00097263_51, and CAP7_c11156_108) or in the late 2000s (e.g., Excalibur_c29304_176, IACX6346, and Ra_c11263_2353) during the breeding program. Of the markers that showed a complete reversal of allelic state, 11% are fixed (allele frequency = 1) in the population (Supplementary Figure S3).

The distribution pattern of markers under selection was different between chromosomes (Figure 3), outliers spanned several genomic regions on the entire chromosome (e.g., 1B, 2B, and 7A) or were clustered at only few localized regions on the chromosome (e.g., 2A and 4A). The markers under selection were clustered into 84 LD-based haplotype loci, containing 1–28 SNPs (Supplementary Table S2). The number of haplotype loci varied among chromosomes, ranging from 3 (chromosomes 2A, 3B) to 11 (chromosome 7A). Thirty-six (30/84) percent of the haplotype loci under selection harbored markers with a complete reversal of allelic state in response to selection over time; the proportion of SNPs with a complete reversal of allelic ranging from 20 (*hap_5A_5*) to 100% (e.g., *hap_1B_5*).

### Phenotypes Analysis

**FIGURE 5:**
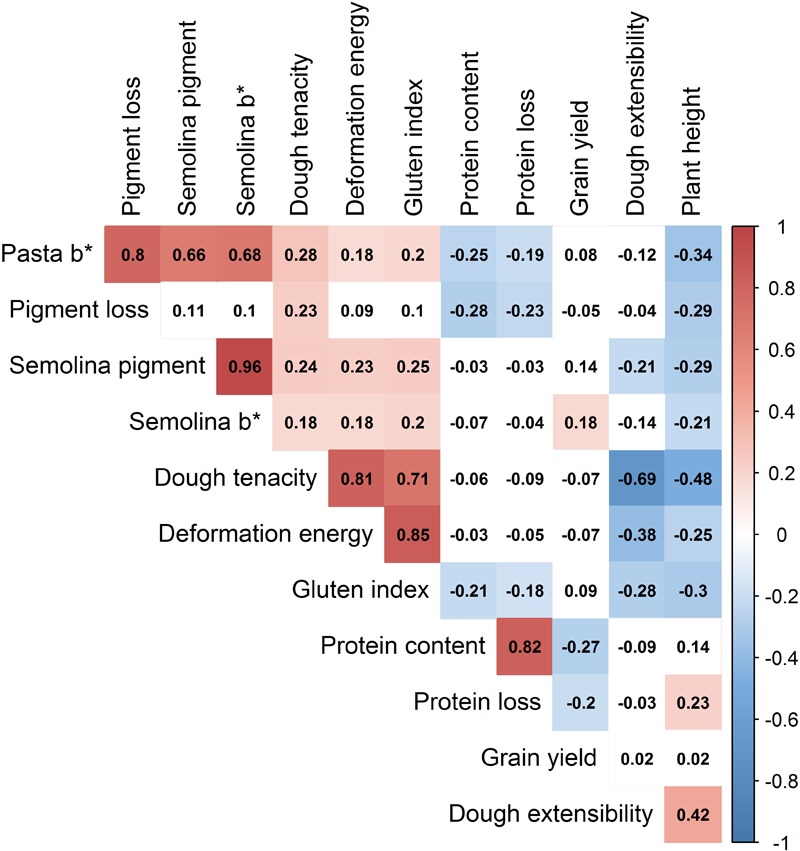
Heatmap of correlation coefficients between all traits measured in the durum wheat breeding panel. The color intensity (red for positive, blue for negative) increases with higher correlation. Absolute values > 0.15 were significant at α = 0.05. ^∗^Should be read as star (e.g., Semolina b^∗^ is “Semolina b star”).

Wide phenotypic variation was observed among lines in the breeding panel for all of the traits (Table 3 and Supplementary Figure S4). Sample means were significantly (*P*-value ≤ 0.05) different between sub-populations for all of the traits, except grain yield. Many traits were significantly (*P*-value ≤ 0.05) correlated to each other (Figure 5). In particular, pasta b^∗^ was strongly positively correlated with pigment loss, semolina pigment and semolina b^∗^, *r* = 0.80, 0.66, and 0.68, respectively. Grain protein and protein loss showed significant (*P*-value < 0.05) positive correlation, *r* = 0.82.

**Table 3:** Comparison of traits means between sub-populations (SP1, SP2, SP3, and SP4).

Traits	Population	SP1	SP2	SP3	SP4	Size^1^
Dough tenacity (P)	78.98 (± 15.00)	85.73^a^	78.81^b^	71.61c	85.59a	168/38/47/45/38
Deformation energy (W)	204.23 (± 41.44)	221.82^a^	204.47^b^	177.16^c^	218.37^ab^	168/38/47/45/38
Dough extensibility (L)	100.23 (± 15.48)	97.10^a^	95.94^b^	111.20^a^	95.70^b^	168/38/47/45/38
Gluten index	63.93 (± 15.17)	69.45^a^	65.20^a^	55.69^b^	66.57^a^	169/38/48/45/38
Semolina pigment	8.34 (± 1.12)	8.27^b^	8.84^a^	7.77^c^	8.60^ab^	184/42/48/54/40
Pasta b^∗^	64.75 (± 1.83)	64.73^b^	64.27^b^	64.55^b^	65.62^a^	168/38/47/45/38
Semolina b^∗^	34.22 (± 1.59)	34.04^ab^	34.65^a^	33.68^b^	34.53^a^	167/38/46/45/38
Pigment loss	-0.07 (± 1.36)	0.32^a^	-0.86^b^	0.10^a^	0.59^a^	168/38/47/45/38
Protein content	13.65 (± 0.27)	13.63^b^	13.78^a^	13.59^b^	13.58^b^	173/39/50/46/38
Protein loss	0.95 (± 0.23)	0.91^b^	1.06^a^	0.93^b^	0.88^b^	173/39/50/46/38
Plant height	89.11 (± 5.04)	88.83^b^	88.78^b^	91.69^a^	86.71^b^	173/39/50/46/38
Grain yield	4088.91 (± 99.41)	4101.94^a^	4098.36^a^	4076.34^a^	4078.34^a^	175/39/50/46/38

*^*1*^Number of lines in the population (entire panel), SP1, SP2, SP3, and SP4, respectively. Values with the same appended letter are not significantly different according to the least significant difference test at p < 0.05 (for each trait). ^∗^Should be read as star (e.g., Pasta b^∗^ is “Pasta b star”).*

### Haplotype-Traits Association Analyses

The association analysis detected 54 haplotype loci, of which 39% (21/54) were associated with at least two traits. For quality traits (Table 4), 49 loci were detected, spanning all chromosomes. In particular, *hap_1B_2* on chromosome 2B was associated with dough extensibility, gluten index, dough tenacity and deformation energy while *hap_4B_2* (chromosome 4B) was associated with pigment loss, pasta b^∗^, protein loss, semolina pigment and protein content. The phenotypic variations explained by the pleiotropic loci varied depending on the traits. For example, *hap_4B_1* explained 20.6, 17.9, 16.6, and 12.1% of the phenotypic variance of pigment loss, pasta b^∗^, dough extensibility and gluten index, respectively, whereas *hap_4B_2* explained 6.8% (semolina pigment, protein content) to 16.8% (protein loss) of the phenotypic variation.

**Table 4:** Haplotypes loci significantly (*P*-value ≤ 0.05) associated with quality traits as revealed by the Mixed Linear Model with Kinship matrix (MLM-K).

Haplotypes	Position^a^	Traits	*P*-value	*R*^2^ (%)	Allelic effect^b^
*hap_1A_1*	1A (1.3–1.7)	Dough extensibility	8.71E-03	9.6	15.94
		Deformation energy	1.42E-02	8.8	30.68
		Semolina pigment	2.11E-02	8.5	1.67
		Gluten index	2.57E-02	7.8	11.7
*hap_1A_3*	1A (58.4–61.2)	Semolina pigment	2.99E-02	7.0	0.39
*hap_1A_4*	1A (116.4–123.2)	Pigment loss	5.35E-02	6.7	-0.2
*hap_1A_6*	1A (149.5–149.5)	Pasta b^∗^	4.36E-02	3.8	3.54
*hap_1B_1*	1B (0.3–5.5)	Protein content	2.24E-02	9.8	1.78
		Dough extensibility	1.82E-02	9.5	22.26
*hap_1B_2*	1B (15.7–15.7)	Dough extensibility	2.68E-04	8.3	2.81
		Gluten index	1.80E-03	6.0	2.8
		Dough tenacity	1.23E-02	3.8	9.73
		Deformation energy	2.42E-02	3.1	6.35
*hap_1B_3*	1B (35.7–38.8)	Semolina pigment	3.51E-02	12.7	1.67
*hap_1B_5*	1B (86.1–96.2)	Semolina pigment	2.52E-02	15.8	1.75
*hap_1B_6*	1B (109–109.8)	Semolina pigment	2.70E-02	5.2	0.27
*hap_1B_7*	1B (115.7–119.1)	Semolina pigment	2.01E-02	13.8	2.39
		Protein content	2.66E-02	12.2	2.25
*hap_1B_10*	1B (158–164.1)	Protein loss	2.52E-02	13.4	-0.54
*hap_2A_1*	2A (107.7–108.9)	Dough extensibility	1.50E-02	13.8	28.58
*hap_2A_3*	2A (181.2–187.7)	Semolina pigment	3.04E-02	15.4	3.17
		Protein loss	3.13E-02	15.3	-0.72
*hap_2B_5*	2B (126.6–131.9)	Semolina pigment	2.08E-02	8.5	1.67
*hap_2B_6*	2B (142.2–146.8)	Semolina pigment	4.23E-02	9.9	1.67
*hap_2B_7*	2B (155.5–158.3)	Gluten index	1.53E-02	7.5	15.95
		Semolina pigment	4.66E-02	6.4	0.72
*hap_2B_9*	2B (181.6–185.3)	Protein loss	1.44E-03	17.8	-0.32
		Protein content	3.84E-03	15.9	2.32
		Deformation energy	2.26E-02	13.0	54.18
*hap_3A_3*	3A (54.7–54.7)	Semolina pigment	1.56E-02	4.7	0.04
*hap_3A_4*	3A (64.2–67.3)	Semolina pigment	5.20E-02	5.3	0.03
*hap_3A_5*	3A (77.8–90.4)	Semolina pigment	4.66E-02	23.5	2.11
*hap_3A_6*	3A (124.6–128.7)	Semolina b^∗^	3.53E-02	9.4	2.19
*hap_3B_2*	3B (144.8–146.4)	Semolina pigment	7.94E-03	11.9	1.67
*hap_4A_2*	4A (64.1–64.1)	Semolina pigment	2.19E-02	5.5	1.86
*hap_4A_3*	4A (90.6–90.6)	Semolina pigment	4.23E-02	3.6	1.67
*hap_4A_4*	4A (118.5–118.5)	Dough tenacity	3.28E-03	5.3	8.71
		Dough extensibility	2.86E-02	2.9	2.75
*hap_4A_6*	4A (173.3–175.8)	Semolina pigment	7.44E-03	8.0	2.86
		Semolina b^∗^	2.74E-02	6.8	3.64
*hap_4B_1*	4B (17.7–22.5)	Pigment loss	1.36E-03	20.6	-0.81
		Pasta b^∗^	5.06E-03	17.9	3.98
		Dough extensibility	9.50E-03	16.6	24.32
		Gluten index	4.52E-02	12.1	21.3
*hap_4B_2*	4B (59.8–60.4)	Pigment loss	2.33E-06	16.8	-1.1
		Pasta b^∗^	3.04E-05	13.3	2.29
		Protein loss	7.72E-04	11.0	-0.53
		Semolina pigment	1.70E-02	6.8	1.28
		Protein content	3.57E-03	6.8	0.93
*hap_4B_4*	4B (105.5–110.2)	Semolina pigment	2.52E-02	9.1	1.67
*hap_5A_1*	5A (43.8–43.8)	Semolina pigment	1.43E-02	4.8	0.23
*hap_5A_2*	5A (50.5–54.9)	Semolina pigment	3.86E-02	5.7	0.59
*hap_5A_3*	5A (64.2–73.6)	Semolina pigment	5.88E-03	15.3	2.86
*hap_5A_5*	5A (140.6–141.4)	Semolina pigment	6.86E-03	11.2	1.86
*hap_5B_1*	5B (7.2–10.1)	Semolina pigment	2.13E-02	7.5	0.27
		Dough extensibility	4.85E-02	5.9	1.26
*hap_5B_4*	5B (181.5–181.5)	Semolina pigment	1.17E-02	5.0	0.15
*hap_6A_3*	6A (39.5–41)	Semolina pigment	2.11E-03	9.7	1.67
		Pasta b^∗^	3.15E-02	5.4	3.54
*hap_6B_2*	6B (36.2–37.8)	Semolina pigment	1.58E-02	8.0	1.3
*hap_6B_3*	6B (54.6–57)	Semolina pigment	3.86E-02	9.3	2.51
*hap_6B_5*	6B (92.3–96)	Dough tenacity	2.03E-02	6.0	3.01
*hap_7A_1*	7A (21.9–21.9)	Semolina pigment	1.67E-02	4.6	0.36
		Protein loss	4.28E-02	3.5	-0.01
*hap_7A_3*	7A (55.9–62.5)	Semolina pigment	2.59E-02	21.3	2.86
*hap_7A_4*	7A (82.4–84.4)	Semolina pigment	2.99E-02	8.8	1.67
*hap_7A_5*	7A (90.9–90.9)	Semolina pigment	4.87E-02	3.4	1.67
*hap_7A_6*	7A (102.4–102.4)	Gluten index	1.53E-02	3.6	0.01
		Protein content	1.53E-02	3.5	0.14
		Dough tenacity	4.70E-02	2.4	6.57
		Deformation energy	5.02E-02	2.3	1.06
*hap_7A_7*	7A (112.2–118)	Semolina pigment	3.60E-02	9.4	1.67
*hap_7A_9*	7A (154.6–158.8)	Semolina pigment	1.81E-02	12.3	1.67
		Deformation energy	3.78E-02	11.9	58.48
*hap_7A_11*	7A (193.9–194.6)	Semolina pigment	6.52E-03	5.7	1.6
		Protein content	1.50E-02	5.0	1.27
		Semolina b^∗^	4.23E-02	3.9	1.62
*hap_7B_3*	7B (120.4–127.4)	Semolina pigment	1.62E-02	11.6	2.51
		Pasta b^∗^	3.48E-02	10.3	3.54
*hap_7B_4*	7B (132.8–133)	Semolina pigment	1.67E-02	4.6	0.33

*^*a*^Position in centiMorgan on the chromosome. ^*b*^Allelic effect: The allelic effect of haplotypes was estimated as the difference between the mean value of the lines carrying these haplotypes and the mean value of the entire population for each trait. ^∗^Should be read as star (e.g., Semolina b^∗^ is “Semolina b star”).*

For protein content, six loci were detected, spanning chromosomes 1B, 2B, 4B, and 7A (2 loci) and explained 3.5 (*hap_7A_6*) to 15.9% (*hap_2B_9*) of the phenotypic variation (Table 4).

Five loci, located on chromosomes 1B, 2A, 2B, 4B, 7A, and 7B, were associated with protein loss (Table 4). In particular, *hap_2B_9, hap_2A_3*, and *hap_1B_10* explained 17.8, 15.3, and 13.4% of the phenotypic variance, respectively.

Semolina pigment showed associated loci on all 14 chromosomes of the durum wheat genome. In particular, haplotype loci *hap_3A_5, hap_7A_3, hap_1B_5, hap_2A_3*, and *hap_5A_3* explained 23.4, 21.2, 15.8, 15.4, and 15.3% of the phenotypic variation, respectively.

For pigment loss, three loci were detected, *hap_1A_4, hap_4B_1*, and *hap_4B_2*, explaining 6.7, 20.6, and 16.8% of the phenotypic variation.

A total of five loci, *hap_1A_1, hap_1B_2, hap_2B_7, hap_4B_1*, and *hap_7A_6* were associated with gluten index. The locus *hap_4B_1* gave the highest allelic effect (21.3) and explained 12.1 % of gluten index variation.

Five loci, located on chromosomes 1A, 4B (2 loci), 6A, and 7B, were significantly (*P*-value ≤ 0.05) associated with pasta b^∗^, explaining 5.4 (*hap_6A_3*) to 17.9% (*hap_4B_1*) of the phenotypic variation.

A total of 11 haplotype loci were associated with plant height and/or grain yield (Table 5). These loci spanned chromosomes 2A, 4A, 4B, 5A, 7A, and 7B for plant height; and 1B, 2B, 4A, and 4B for grain yield. In particular, the loci *hap_4A_5* and *hap_4B_1* controlled both plant height and grain yield. The locus *hap_4A_5*, which showed the highest allelic effect (190.03) on grain yield, explained 43.9 and 38.5% of the phenotypic variation of plant height and grain yield, respectively. On the other hand, *hap_4B_1* was strongly associated (*r*^2^ = 0.90) with the dwarfing gene *Rht-B1b* and explained 30.0% of the phenotypic variation of plant height.

**Table 5:** Haplotype loci significantly (*P*-value ≤ 0.05) associated with plant height and grain yield as revealed by the Mixed Linear Model with Kinship matrix (MLM-K).

Haplotypes	Position^a^	Trait	*P*-value	R^2^ (%)	Allelic effect^b^
*hap_1B_8*	1B (128.8–128.8)	Grain yield	3.28E-02	5.2	28.66
*hap_2A_1*	2A (107.7–108.9)	Plant height	6.07E-03	15.0	-9.71
*hap_2B_2*	2B (19–19)	Grain yield	4.45E-03	6.5	5.95
*hap_2B_7*	2B (155.5–158.3)	Grain yield	2.29E-04	13.4	76.52
*hap_4A_5*	4A (148.3–167.6)	Plant height	1.04E-02	43.9	-3.15
		Grain yield	4.03E-02	38.5	190.03
*hap_4A_6*	4A (173.3–175.8)	Grain yield	9.49E-04	11.4	5.34
*hap_4B_1*	4B (17.7–22.5)	Plant height	6.44E-06	30.0	-9.09
		Grain yield	2.25E-02	14.4	65.21
*hap_4B_2*	4B (59.8–60.4)	Plant height	1.01E-06	17.4	-8.03
*hap_5A_1*	5A (43.8–43.8)	Plant height	4.74E-02	2.3	-0.29
*hap_7A_10*	7A (168.6–168.6)	Plant height	1.61E-03	6.0	-2.73
*hap_7B_5*	7B (147–147)	Plant height	4.90E-02	3.6	-3.48

*^*a*^Position in centiMorgan on the chromosome. ^*b*^Allelic effect: The allelic effect of haplotypes was estimated as the difference between the mean value of the lines carrying these haplotypes and the mean value of the entire population for each trait.*

Thirty-nine (21/54) percent of haplotype loci associated with the traits we investigated contained markers with a complete reversal of allelic state (Supplementary Table S3). In particular, *hap_1B_5* and *hap_2A_1* consisted of 100% (7/7) and 91% (10/11) markers with a complete reversal of allelic state, respectively. For each haplotype locus, the time at which the switch in allelic state occurred varied among SNPs, regardless of the number of traits the haplotype locus was associated with. For example, for *hap_1B_5* associated with only semolina pigment, the switch in allelic state occurred in the early 1960s (Tdurum_contig85180_99), in the late 1980s (BobWhite_c17644_456) and in the 2000s (IACX6346) (Supplementary Figure S5). For *hap_2A_1* that was associated with plant height and dough extensibility, the switch in allelic state occurred in the 1970s (BS00097263_51), in the 1985s (e.g., CAP7_c239_267, CAP7_c11156_108), and in the 1990s (wsnp_Ex_rep_c66615_64916512) (Supplementary Figure S6).

## Discussion

### Population Stratification and Genetic Diversity Over Time

The presence of genetic structure within a population can lead to spurious association signals (Marchini et al., 2004; Kang et al., 2008; Myles et al., 2009; Mezmouk et al., 2011; Cappa et al., 2013). Therefore, the analysis of the actual population structure of the durum breeding panel was intended to limit the FDR in the association analysis. The discriminant analysis of principal components (Jombart et al., 2010) clustered the 192 lines into four sub-populations. Nonetheless, the genetic differentiation between the four sub-populations was moderate (0.05 < Fst ≤ 0.15), whereas 85.2% of the genetic variation resided between individuals. Therefore, the association analysis accounted only for kinship (MLM-K model). The population structure is in agreement with known differences in pedigree, breeding program sources and era of testing in the trials, as described in our previous study (N’Diaye et al., 2017). Similarly, many other studies about genetic diversity changes in durum wheat germplasm over time revealed that most (up to 91%) of the variations were found between individuals within groups compared to that between groups (Henkrar et al., 2016).

Durum breeding began in Canada in the early 1950s, with the first locally-bred cultivar registered in 1963. The Canadian wheat industry has strict processing quality requirements for registration of durum cultivars, so the majority of breeding crosses involve closely-related local materials, with relatively little use of either introduced modern cultivars or landraces except where absolutely necessary to introduce a new trait. Genetic diversity in the registration trials changed over time, declining during the first 20 years of breeding in Canada when the germplasm shifted from introduced cultivars in the 1940s and 1950s to locally-bred lines in the 1970s (Figure 1). Diversity increased in the late 1980s and early 1990s coinciding with introgression of higher yellow pigment concentration, gluten strength and low grain cadmium concentration (Clarke J.M. et al., 2010), and with major expansion of Canadian durum breeding programs due to increased funding. The sources of these traits were improved cultivars from CIMMYT, Germany, Italy, and United States. Further cycles of crossing and selection then reduced diversity observed during the past decade to a level similar to the 1970s. In contrast, a relatively stable diversity over 25 years was observed in the CIMMYT Elite Spring Wheat Yield Trial (Dreisigacker et al., 2012), probably reflecting consistent usage of diverse parents to meet requirements for adaptation to global production environments. Similarly, the impact of traits improvement on allelic changes over time has been described in Canadian bread wheat cultivars (Fu and Somers, 2011).

The impacts of modern plant breeding on crop genetic diversity has been a major concern of many scientists in search for implementing efficient conservation strategies and a better utilization of germplasms (see Duvick, 1984; Fu, 2007, 2015 for review). In particular, some temporal patterns of genetic diversity have been observed (Rauf et al., 2010; van de Wouw et al., 2010b,c). The high selection pressure in modern plant breeding has reduced crops genetic diversity over time (Duvick, 1984; Hallauer and Darrah, 1985; Bowman, 2003; Gepts, 2006; Fu and Dong, 2015). In particular, allelic reduction at specific loci and allele loss over time have been reported in the Canadian hard red spring wheat germplasm (Fu et al., 2005). However, no loss of allele’s number was found in European winter wheat varieties over time (Huang et al., 2007). Similarly, a meta-analysis of 44 diversity studies did not reveal evidence of genetic erosion in released varieties (van de Wouw et al., 2010a). The discrepancy between genetic diversity trends in released cultivars and varieties was attributed to differences in breeding goals and methods that have been applied over time (Gepts and Hancock, 2006; Rauf et al., 2010). Many other sources that could affect the crops genetic diversity have been reported, including use of different genetic diversity measures, sampling bias and arbitrary grouping of cultivars to represent specific breeding periods (Reif et al., 2005; Fu, 2007; Aremu, 2011).

### Loci Under Selection

Loci under directional selection are expected to have lower intra-population variability and larger inter-population variability than neutral loci. Thus, loci under directional selection can be unveiled by patterns in heterozygosity and/or differences in Fst values (Eveno et al., 2008; Perez-Figueroa et al., 2010; Kirk and Freeland, 2011; Konijnendijk et al., 2015; Lin et al., 2017). Evidence for selection was investigated using, the total variance Fst-based outlier detection method implemented in Lositan (Antao et al., 2008), the hierarchical island model implemented in Arlequin (Excoffier and Lischer, 2010) and the Bayesian genome scan approach implemented in BayeScan (Foll and Gaggiotti, 2008). The number of outliers varied according to the method. Lositan detected 403 outliers, including all 397 markers from Arlequin. BayeScan identified the less number (144) of outliers of which 4 SNPs were undetected by Lositan and BayeScan. Comparison of several outlier detection methods showed that these methods differ in their type I (false positives) and type II (false negatives) error rates (Perez-Figueroa et al., 2010; Narum and Hess, 2011). Thus, combining the properties of different outlier detection methods could reduce the percentage of false positives and strengthen the candidate status of the identified outlier loci (Vasemagi et al., 2005). A similar approach has been reported for many crops, including wheat (Gao et al., 2017; Ren et al., 2017), rice (Xia et al., 2014), and apple (Khan et al., 2014). In general, researchers feel more comfortable when loci under selection are detected by at least two different methods. However, among the four outliers detected by only BayeScan, one marker representing the haplotype locus *hap_3A_3*, showed strong association with semolina pigment and explained 4.2% of the phenotypic variance (Table 4). Studies where only one method was used to scan for loci under selection have been reported elsewhere (Mäkinen et al., 2008; Jiao et al., 2012; Liao et al., 2013). Therefore, it seems reasonable to take into consideration any outlier detected by a single method even if it might not be ranked as a prime candidate.

Markers under selection showed different distribution patterns, spanning several genomic regions or clustered at only a few localized regions on the chromosome, reflecting linkage and/or pleiotropy. It is now well documented that genes are not evenly distributed across the genome and QTL might be clustered in ‘hot regions’. The clustering of QTL for different traits have been reported for many crops, including rice (Cai and Morishima, 2002; Crowell et al., 2016; Singh et al., 2017), wheat (Zhang et al., 2007, 2015), eggplant (Portis et al., 2014), cabbage (Lv et al., 2016), and Brassica (Basnet et al., 2015). The existence of such hot regions could offer an appealing opportunity to select for multiple traits, especially when traits are positively correlated.

Thirty-six (30/84) percent of the haplotype loci under selection harbored markers with a complete reversal of allelic state. This tendency towards fixation of favorable alleles in our material corroborates the success of the Canadian durum wheat breeding programs over time.

### Genome-Wide Association Studies

Previous studies showed that haplotype-based analysis increases the power of QTL detection (Liu et al., 2008; Hamblin and Jannink, 2011; Gawenda et al., 2015; Contreras-Soto et al., 2017) and explains a larger proportion of the QTL variance (Grapes et al., 2006; Hayes et al., 2007; N’Diaye et al., 2017) as compared to single-marker analysis. Therefore, all of the 407 SNP markers under selection were clustered into 84 LD-based haplotype loci, as reported in our previous manuscript (N’Diaye et al., 2017).

Thirty-six (30/84) percent of the haplotype loci under selection failed to be associated with any of the traits that we investigated. Because these loci showed strong evidence of directional selection, the lack of an association signal could suggest that they might be controlling traits that we do not have data for, and we didn’t analyze here. Therefore, these loci could still be of interest for further investigations when more phenotypic data becomes available.

A total of 49 haplotype loci, spanning all 14 chromosomes of the durum wheat genome, were associated with quality traits. In particular, *hap_4B_1* located on chromosome 4B explained 20.6, 17.9, 16.6, and 12.1% of the phenotypic variance of pigment loss, pasta b^∗^, dough extensibility and gluten index, respectively. This result is congruent with many studies that reported major QTL for yellow pigment on chromosome 4B (Mares and Campbell, 2001; Pozniak et al., 2007; Blanco et al., 2011; Roncallo et al., 2012; N’Diaye et al., 2017). The haplotype locus *hap_3A_5* explained 23.4% of the phenotypic variation of semolina pigment, contrasting with the minor QTL of pigment color reported by other studies in durum wheat (Reimer et al., 2008; Roncallo et al., 2012). However, major QTL explaining up to 20% of the variation of flour color have been reported on chromosome 3A in hexaploid wheat (Parker et al., 1998; Mares and Campbell, 2001; Crawford and Francki, 2013). The locus *hap_7A_3* explained 21.3% of the phenotypic variation of semolina pigment; coincident with the existence of a major QTL of yellow pigment on chromosome 7A in durum wheat (Patil et al., 2008; Zhang and Dubcovsky, 2008; Zhang et al., 2008). These conflicting results clearly demonstrate the complex inheritance of yellow pigment in wheat.

Protein content is a key specification for wheat because of its nutritive value and its impact on many processing properties, such as water absorption and gluten strength (Delcour et al., 2010; Kaur et al., 2015; Kaushik et al., 2015). Six haplotype loci were associated with protein content, spanning different chromosomes, of which chromosome 6B that had been reported to harbor a major QTL for grain protein (Joppa et al., 1997; Olmos et al., 2003; Uauy et al., 2006; Patil et al., 2009; Suprayogi et al., 2009; Conti et al., 2011). Nonetheless, in our study the highest proportion (15.9%) of variance explained came from the locus *hap_2B_9*, located on chromosome 2B. Depending on the populations, the QTL explaining the largest variance of protein content in durum wheat was found on different chromosomes, such as 2A (Blanco et al., 2006), 5B (Golabadi et al., 2012), and 5A (Marcotuli et al., 2017). Similarly, in hexaploid wheat, the number of QTL controlling protein content were reported on different chromosomes (Börner et al., 2002; Sun et al., 2008; Wang et al., 2012; Li et al., 2013; Terasawa et al., 2016; Tiwari et al., 2016; Zou et al., 2017). These results suggest the complex inheritance of grain protein and the strong influence of the underlying population and the environment.

High semolina protein content determines end-use products quality such as texture, appearance and firmness. However, because of its negative correlation with grain yield (Blanco et al., 2006; Würschum et al., 2016), selecting for high grain yield has indirectly resulted in lines with lower protein content (De Vita et al., 2007; Acreche and Slafer, 2009). To simultaneously improve grain yield and protein content in durum wheat, an index selection method has recently been developed (Rapp et al., 2018). Moreover, it has been reported that protein content substantially decreases (up to 25% loss of protein) during milling mainly with regard to the milling methods (Prabhasankar and Haridas Rao, 2001; Ramberg and McAnalley, 2002; Heshe et al., 2016; Oghbaei and Prakash, 2016). Because the concentrations of protein components follow a gradient (higher concentration in the outer layer) along the different parts of the grain (Tosi et al., 2011; He et al., 2013; Li et al., 2016; Savill et al., 2018) and that this gradient is in part determined by genetic factors (He et al., 2013), one might wonder if protein loss during milling could have some genetic basis. To the best of our knowledge, no investigation for a possible genetic basis of protein loss has been carried out. In this study, five haplotype loci were associated with protein loss, explaining 3.5 (*hap_7A_1*) to 17.8% (*hap_2B_9*) of the phenotypic variance. The haplotype locus *hap_2B_9*, explaining the highest proportion (17.8%) of protein loss variance, also explained 15.9% of the variation of protein content. This result is not surprising given the strong positive (*r* = 0.82) correlation between these two traits; the higher protein content the bigger the protein loss. Similarly, Savill et al. (2018) reported that any increase in the gradient of protein concentration may result in an even greater amount of protein being lost during milling. In fact, during milling some endosperm tissues remain adhered to the bran layers and because protein is concentrated in the outer layers of the endosperm, a proportionally greater amount of protein relative to starch is lost during the production of white flour. Therefore, there is a need to select for genotypes having high protein content but low protein loss during milling. The availability of many haplotype loci for both protein content and protein loss offers the opportunity to screen different allele combinations to work this challenge out.

A total of 11 haplotype loci were associated with plant height and/or grain yield, explaining low (2.3%) to high (43.9%) proportion of the phenotypic variance depending on the trait. For grain yield, of the six haplotype loci that were detected, *hap_4A_5* located on chromosome 4A, explained 38.5% of the phenotypic variance. Yield is the most important and genetically complex trait in wheat, being controlled by a large number of small effect QTL across all chromosomes (Wu et al., 2012). Many QTL and marker-trait associations for yield and yield-related traits (e.g., spike length, spikelet per spike and 1000 kernel weight) have been reported on all chromosomes of wheat (Marza et al., 2006; Heidari et al., 2011; Ma et al., 2012; Li et al., 2015; Marcotuli et al., 2017). The major yield QTL were reported on different chromosomes depending on the study. In particular, a comprehensive QTL analysis in a durum wheat population across 16 environments detected 16 QTL of grain yield, including two major QTL on 2B and 3B, explaining 21.5 and 13.8% of the variance, respectively (Maccaferri et al., 2008). Nonetheless, of the many QTL regions affecting yield and yield-related traits, two strong QTL hotspots were identified on chromosomes 2A and 2B in a durum wheat panel of 208 lines (Sukumaran et al., 2018). In contrast, the most important QTL affecting yield in a durum wheat population evaluated in different environments were reported on chromosome 3B and 3A, explaining 20.7 and 18.0 % of the phenotypic variation, respectively (Roncallo et al., 2017). A number of QTL controlling grain yield have also been reported on different chromosomes of common wheat (Quarrie et al., 2006; Zhang et al., 2010; Wang et al., 2012; Simmonds et al., 2014; Li et al., 2015; Tahmasebi et al., 2016; Sehgal et al., 2017). In particular, one and two major yield QTL were reported on chromosomes 3A (Mengistu et al., 2012) and 3B (Bennett et al., 2012), respectively.

Among the seven haplotype loci associated with plant height, *hap_4A_5* (chromosome 4A) and *hap_4B_1* (chromosome 4B) explained 43.9 and 30.0% of the variance, respectively. The *Rht-B1b* gene was mapped on chromosome 4B. These results are consistent with those of many studies that reported major QTL for plant height on chromosomes group 4 in both durum and hexaploid wheat (Gao et al., 2015; Guo et al., 2017; Iannucci et al., 2017; Shi et al., 2017). Moreover, many QTL and marker-trait associations for plant height have been reported, spanning all chromosomes of wheat (Griffiths et al., 2012; Bentley et al., 2014; Zanke et al., 2014). Plant height is one of the most studied traits in wheat because it determines the general architecture of the plant and has strong effect on lodging stability, harvest index and ultimately grain yield. Because tall plants are more susceptible to lodging (Berry et al., 2003), plant height reduction has been a major target for wheat breeding programs for many years. As a result, major dwarfing genes such as *Rht-D1b, Rht-B1b, Rht8*, and *Rht12* have been incorporated in new wheat varieties to reduce plant height without reducing grain yield potential (Flintham et al., 1997; Korzun et al., 1998; Ellis et al., 2002; Borojevic and Borojevic, 2005; Kowalski et al., 2016).

The haplotype locus *hap_4A_5* was associated with both plant height and grain yield, explaining 43.9 and 30.0% of the phenotypic variation, respectively. The influence of plant height on grain yield is well known and pleiotropic loci controlling these two traits have been reported in durum wheat (Maccaferri et al., 2008) and common wheat (McCartney et al., 2005; Gao et al., 2015; Cabral et al., 2018).

Overall, the presence of numerous pleiotropic haplotype loci, such as *hap_2B_9* (protein content, protein loss, deformation energy), *hap_4A_5* (plant height, grain yield) and *hap_4B_1* (pigment loss, pasta b^∗^, dough tenacity, gluten index), offers breeders the appealing opportunity for improving multiple traits. Indeed, pinpointing the genes controlling these traits and identifying causal mutations would be greatly resourceful to the wheat community. However, tracking down causative genes is not trivial and might take several years, especially when dealing with many complex traits. For sake of information, we retrieved the genes and their annotations (Supplementary Table S4), using the Chinese spring reference genome (Appels et al., 2018).

Thirty-nine percent of haplotype loci associated with the traits we investigated contained markers with a complete reversal of allelic state, suggesting a tendency of fixation of favorable alleles in our plant material. However, according to the theory of selection limits in finite population such as plant breeding populations, artificial selection is expected to increase the frequency of favorable alleles, with the possibility of other less desirable or selectively neutral alleles being fixed simply by chance (Robertson, 1960). Long-term improvement of a trait influences genes associated with the trait and nearby chromosomal regions via linkage (Hedrick, 2000). Therefore, separating the loci directly controlling the improved trait from those derived from hitchhiking could be challenging (Fu and Somers, 2011). Thus, for each haplotype locus, we only kept the alleles series offering the largest allelic effect on the phenotype.

The time at which the switch in allelic state occurred varied among SNPs within each haplotype locus, probably due to the involvement of different genes in the genetic control of the targeted traits. The selection response of complex traits is the result of simultaneous changes in allele’s frequencies across many genetic variants and large effect loci are more likely to be fixed rapidly (Johansson et al., 2010). However, because of the relatively small size of our population (192 lines) and the possible involvement of many genes with small effects, it is challenging to predict how fast the favorable alleles will be fixed. Similarly, many years (1845–2004) of improvement of different traits in Canadian hard red spring wheat introduced significant changes in allele frequencies at different loci across the whole genome (Fu and Somers, 2011).

Our results clearly demonstrate the impact of artificial selection on the dynamics of the durum wheat genome, in terms of allelic changes at selected loci over time. The long-term breeding efforts have impacted different chromosomal regions as many genes were targeted by the selection of complex traits.

## Conclusion

Eighty-four LD-based haplotype loci showed strong evidence of selection over 60 years of breeding in Canadian durum wheat germplasm. The distribution pattern of these loci was different between chromosomes, outliers spanned several genomic regions on the entire chromosome (e.g., 1B, 2B, and 7A) or were clustered at only few localized regions on the chromosome (e.g., 2A and 4A). The association analysis detected 54 haplotype loci, of which 39% contained markers with a complete reversal of allelic state. This tendency towards fixation of favorable alleles corroborates the success of the Canadian durum wheat breeding programs over time. All of the loci under selection uncovered by this study could be helpful in the identification of genes related to many traits of interest as functional annotations of the wheat genome become available, and to facilitate marker-assisted selection in durum wheat.

## Author Contributions

AN designed the experiment, performed all analyses and wrote the manuscript. JKH contributed to the initial draft and edited the manuscript. KTN and SW edited the manuscript. AKS, FRC, JMC, and YR collected phenotypic data and edited the manuscript. CJP supervised the project, designed the experiment, collected phenotypic data and edited the manuscript.

## Conflict of Interest Statement

The authors declare that the research was conducted in the absence of any commercial or financial relationships that could be construed as a potential conflict of interest.

## Supplementary Material

FIGURE S1 Scatter plots showing the thresholds to declare loci being under selection, 0.2 (Arlequin, Lositan) and 0.15 (BayeScan).

FIGURE S2 Q-Q (quantile-quantile) plot profiles of the association analyses using: **(A)** the Mixed Linear Model with the Kinship matrix alone (MLM-K), and **(B)** with both Q matrix from the discriminant analysis of principal component and Kinship (MLM-QK).

FIGURE S3 Distribution of allele’s frequency for the 95 SNP markers having a complete reversal of allelic state. Brown color is the overlap between blue bar (A allele) and orange bar (B allele).

FIGURE S4 Histograms of the phenotypic traits evaluated in the Canadian durum wheat germplasm.

FIGURE S5 Example of changes in allelic states of markers at different time periods for the haplotype locus *hap_1B_5*, associated with only semolina pigment.

FIGURE S6 Example of changes in allelic states of markers at different time periods for the haplotype locus *hap_2A_1*, associated with plant height and dough extensibility.

TABLE S1 Lines pedigree and year of introduction into the Durum wheat Cooperative Test.

TABLE S2 Haplotype loci under selection in the Durum wheat Cooperative Test.

TABLE S3 Haplotype loci associated with traits and containing SNPs with complete reversal of allelic state.

TABLE S4 List of genes harboring outliers associated with phenotypic traits in the durum wheat breeding panel.
