# Supplementary figures and images for: Haplotype Loci Under Selection in Canadian Durum Wheat Germplasm Over 60 Years of Breeding: Association With Grain Yield, Quality Traits, Protein Loss, and Plant Height
Source: Front Plant Sci. 2018 Nov 5;9:1589. doi: 10.3389/fpls.2018.01589 (PMC6230583; doi:10.3389/fpls.2018.01589)

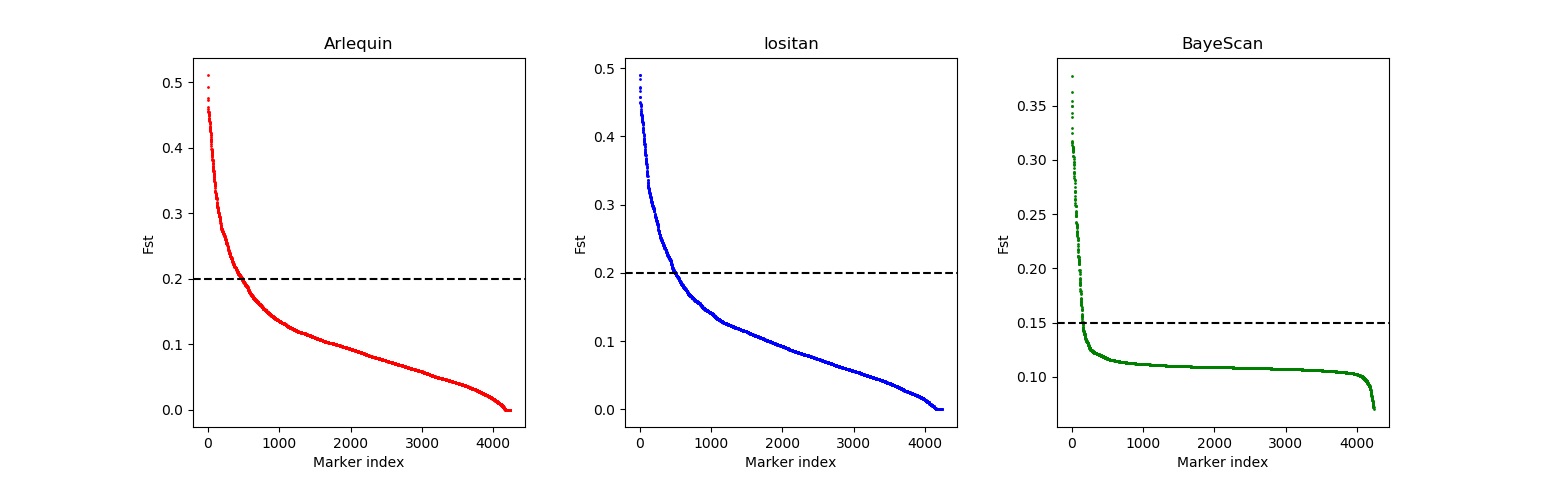

Supplement: FIGURE S1 — Scatter plots showing the thresholds to declare loci being under selection, 0.2 (Arlequin, Lositan) and 0.15 (BayeScan). [file Image_1.JPEG]

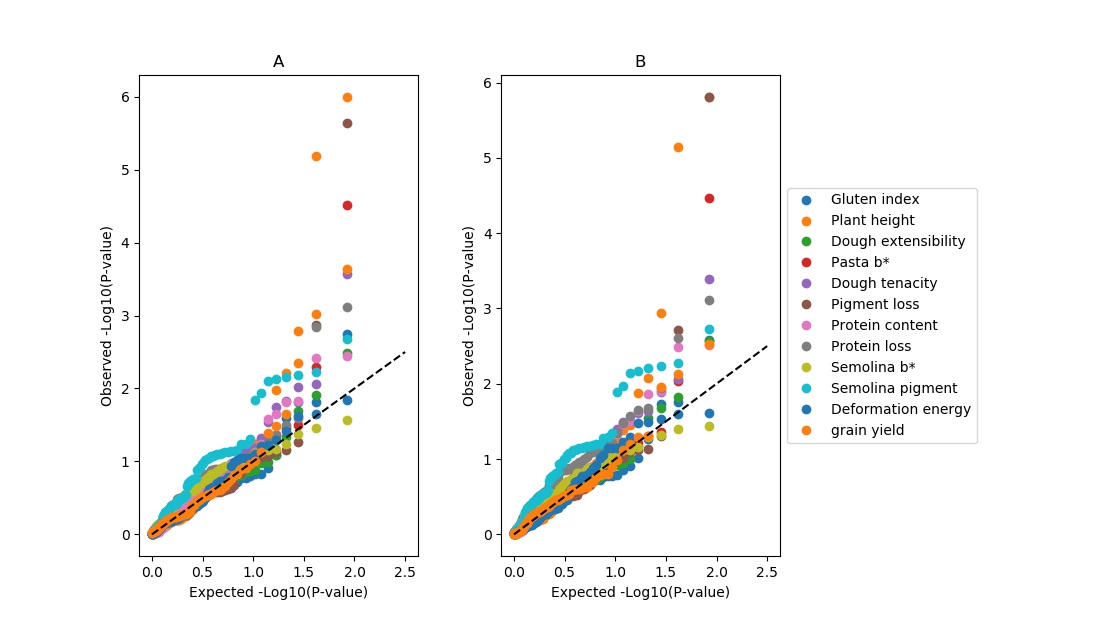

Supplement: FIGURE S2 — Q-Q (quantile-quantile) plot profiles of the association analyses using: (A) the Mixed Linear Model with the Kinship matrix alone (MLM-K), and (B) with both Q matrix from the discriminant analysis of principal component and Kinship (MLM-QK). [file Image_2.JPEG]

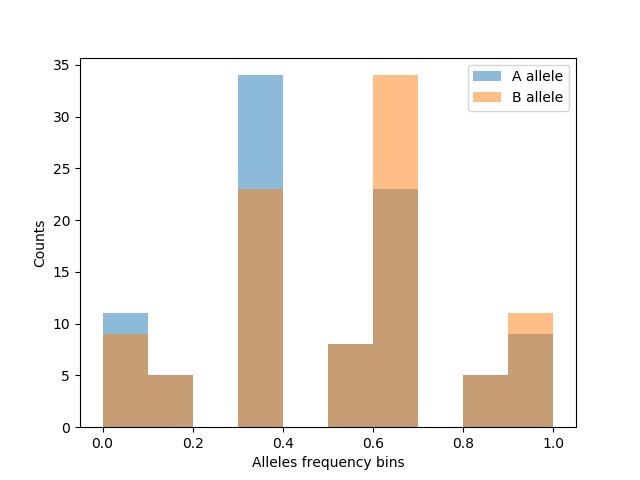

Supplement: FIGURE S3 — Distribution of allele’s frequency for the 95 SNP markers having a complete reversal of allelic state. Brown color is the overlap between blue bar (A allele) and orange bar (B allele). [file Image_3.JPEG]

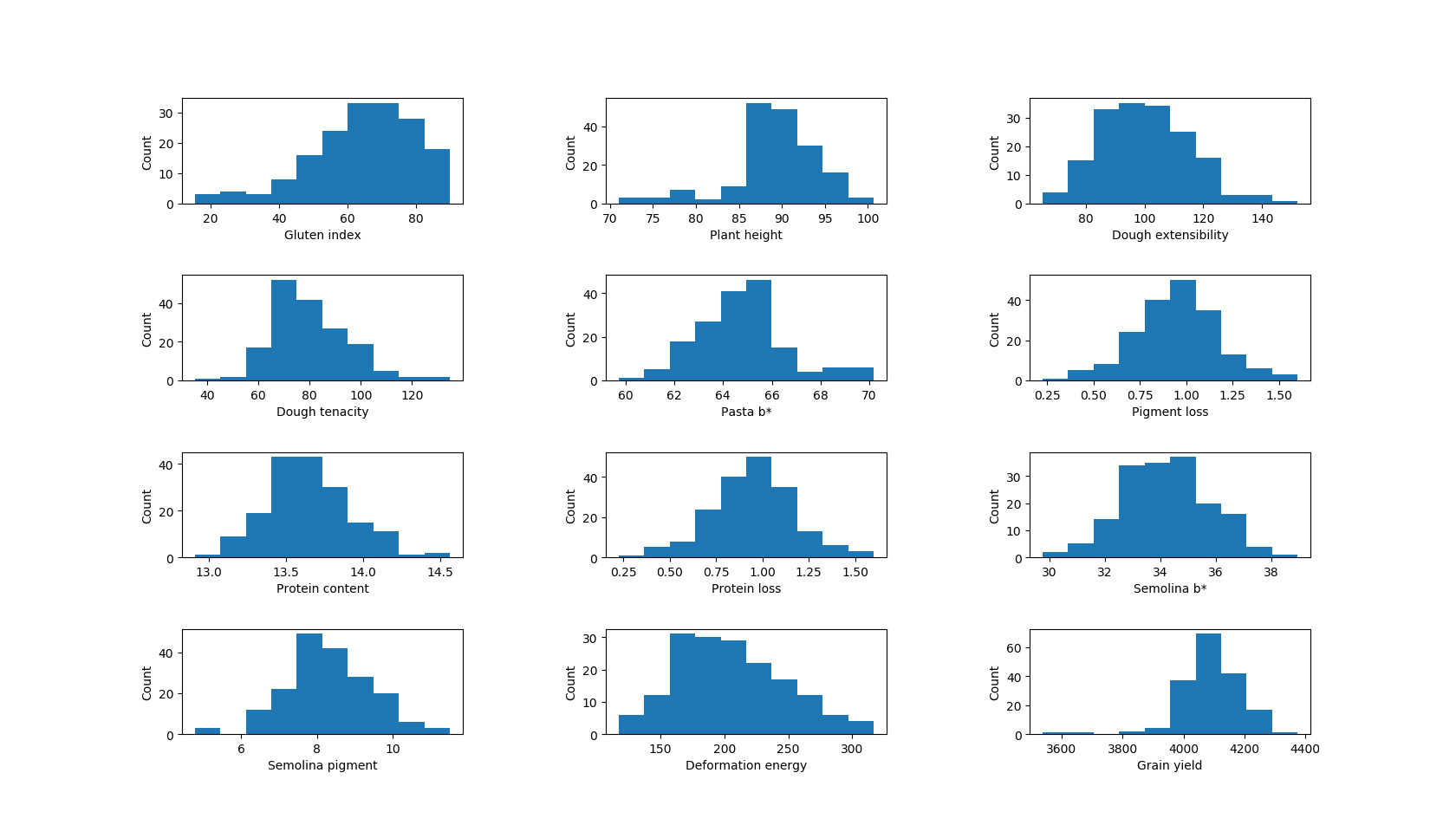

Supplement: FIGURE S4 — Histograms of the phenotypic traits evaluated in the Canadian durum wheat germplasm. [file Image_4.JPEG]

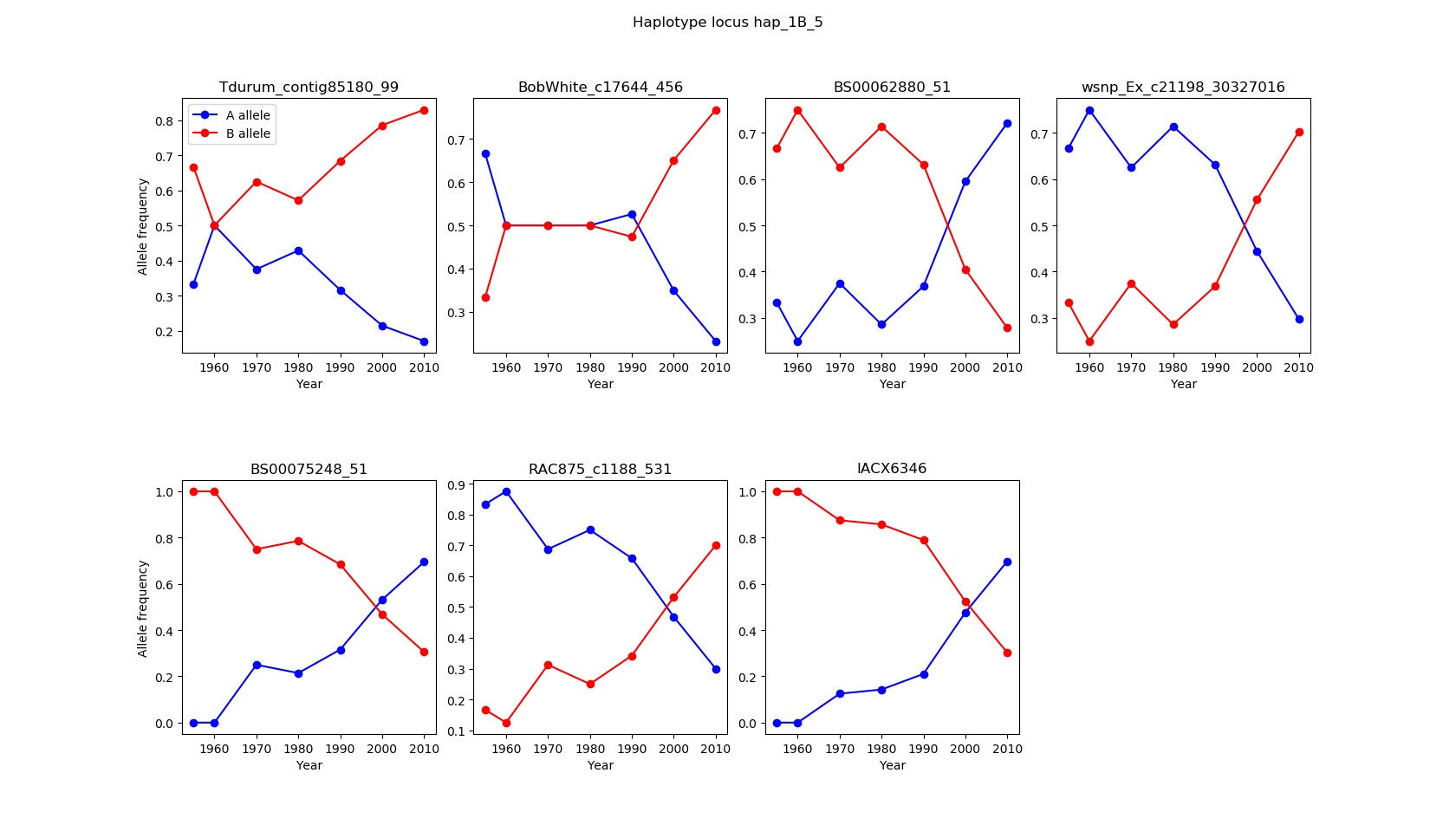

Supplement: FIGURE S5 — Example of changes in allelic states of markers at different time periods for the haplotype locus hap_1B_5, associated with only semolina pigment. [file Image_5.JPEG]

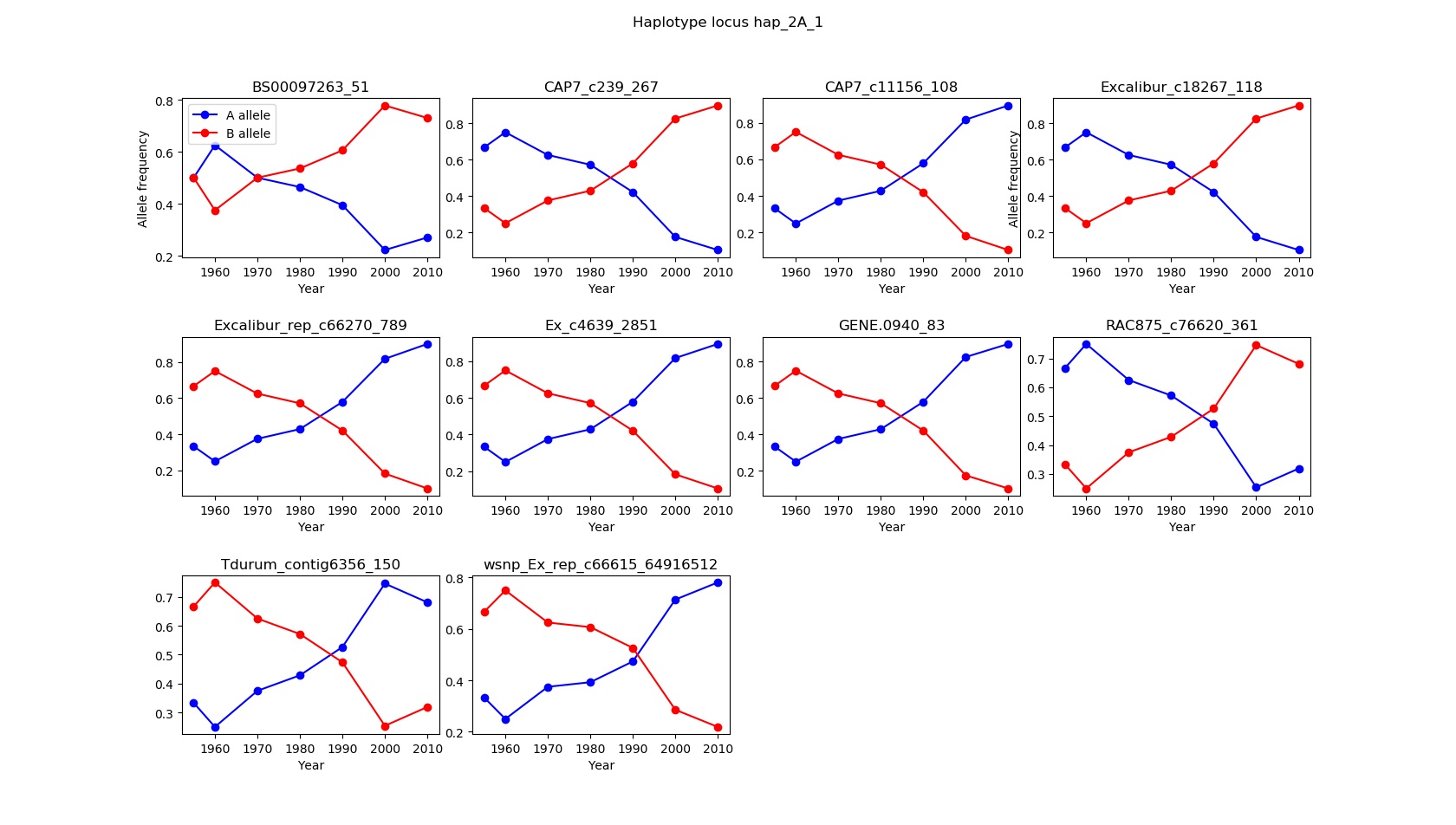

Supplement: FIGURE S6 — Example of changes in allelic states of markers at different time periods for the haplotype locus hap_2A_1, associated with plant height and dough extensibility. [file Image_6.JPEG]
