# Supplementary material for: Haplotype Loci Under Selection in Canadian Durum Wheat Germplasm Over 60 Years of Breeding: Association With Grain Yield, Quality Traits, Protein Loss, and Plant Height
Source: Front Plant Sci. 2018 Nov 5;9:1589. doi: 10.3389/fpls.2018.01589 (PMC6230583; doi:10.3389/fpls.2018.01589)
Supplement: TABLE S1 — Lines pedigree and year of introduction into the Durum wheat Cooperative Test. [file Table_1.DOCX]

**Supplemental Table 1.** Lines pedigree and year of introduction into the Durum wheat Cooperative Test.

| Lines | Pedigree | Year | Sub-population* |
| --- | --- | --- | --- |
| MINDUM | Selection from Hedgreow | 1946 | SP3 |
| PELISSIER | Algerian Landrace | 1946 | SP4 |
| LANGDON | Yuma/Stewart//Carleton | 1955 | SP3 |
| LAKOTA | SENTRY:DR//LD379/LD357 | 1957 | SP3 |
| STEWART63 | STEWART*8/ST.464 | 1959 | SP3 |
| HERCULES | LD308/LD368//STEWART,USA/RL3380 | 1964 | SP3 |
| WAKOOMA | LAKOTA*2/PELISSIER | 1967 | SP4 |
| WASCANA | LAKOTA*2/PELISSIER | 1967 | SP1 |
| MACOUN | RL3607/DT182 | 1970 | SP3 |
| WARD | LDN/3/LD357//CI7780/LD362/4/ISWRN-21 | 1971 | SP3 |
| COULTER | DT188/DT224//DT182 | 1972 | SP3 |
| QUILAFEN | YAKTANA 54/NORIN 10 BREVOR//SENTRY:DR/2*TC | 1973 | SP1 |
| VIC | EDMORE/WARD | 1978 | SP3 |
| DT367 | S-017/WASCANA// 7168 | 1979 | SP1 |
| MEDORA | WARD//RL3607/DT182 | 1979 | SP3 |
| DT369 | WASCANA/QUILAFEN | 1980 | SP1 |
| KYLE | 6962-92-8-5/ 6965-494-1 | 1981 | SP4 |
| SCEPTRE | D72110/COULTER | 1982 | SP3 |
| Lloyd | CANDO/EDMORE | 1983 | SP3 |
| PLENTY | ND74112/WASCANA//DT354 | 1986 | SP3 |
| AC MELITA | MEDORA/LLOYD | 1991 | SP3 |
| AC MORSE | RL 7196/DT610 | 1993 | SP3 |
| AC AVONLEA | 8267-AD2A/DT612 | 1994 | SP2 |
| DT486 | VIC/DT384//DT471 | 1994 | SP3 |
| DT488 | RL7174/SCEPTRE | 1994 | SP3 |
| DT666 | 8262-Y2A/ 8264-F5D | 1995 | SP3 |
| DT668 | 8262-Y2A/ 8264-F5D | 1995 | SP3 |
| DT683 | DT379/DT618//DT474 | 1995 | SP3 |
| AC NAVIGATOR | KYLE/WESTBRED 881 | 1996 | SP4 |
| NAPOLEON | VIC/DT384//DT471 | 1996 | SP3 |
| PATHFINDER | WESTBRED 881/DT367 | 1996 | SP1 |
| DT513 | DT625/DT612 | 1997 | SP3 |
| BEN | D-8024/MONROE | 1998 | SP3 |
| DT518 | DT634/DT627 | 1998 | SP3 |
| DT520 | DT633/DT612 | 1998 | SP3 |
| DT521 | DT618/DT616 | 1998 | SP1 |
| DT684 | DT379/DT618//DT474 | 1998 | SP3 |
| DT685 | 8982-BA5/KYLE | 1998 | SP4 |
| DT687 | DT627/2*KYLE | 1998 | SP3 |
| DT522 | DT636/DT641 | 1999 | SP3 |
| DT523 | D89052/DT644 | 1999 | SP3 |
| DT524 | D90056/D90302 | 1999 | SP3 |
| DT688 | DT618/DT642//DT637 | 1999 | SP3 |
| DT689 | DT618/DT642//DT637 | 1999 | SP3 |
| DT691 | DT618/DT642//DT637 | 1999 | SP3 |
| DT692 | 8667-D037A/ 8960-ADV//DT639 | 1999 | SP3 |
| DT693 | DT639/DT637//DT639 | 1999 | SP3 |
| DT694 | DT639/DT637//DT639 | 1999 | SP3 |
| DT695 | DT471/2*KYLE | 1999 | SP3 |
| DT696 | DT618/DT637//KYLE | 1999 | SP1 |
| DT698 | 8982-TL05/DT662 | 1999 | SP1 |
| DT699 | DT618/DT642//DT637 | 2000 | SP3 |
| DT700 | 8667-D037A/ 8960-ADV//DT639 | 2000 | SP3 |
| DT701 | 8982-BA5C/DT618 | 2000 | SP1 |
| DT702 | 8982-TL05/DT662 | 2000 | SP1 |
| DT703 | 8982-TL05/DT662 | 2000 | SP1 |
| DT704 | AC AVONLEA/DT665 | 2000 | SP2 |
| DT705 | AC AVONLEA/DT665 | 2000 | SP1 |
| DT706 | AC AVONLEA/DT665 | 2000 | SP2 |
| DT707 | AC AVONLEA/DT665 | 2000 | SP2 |
| DT708 | DT674/DT665 | 2000 | SP4 |
| DT709 | DT674/DT665 | 2000 | SP4 |
| DT710 | DT618/GREEN_27 | 2000 | SP1 |
| DT711 | WESTBRED 881/W9260-BK03 | 2000 | SP1 |
| STRONGFIELD | AC AVONLEA/DT665 | 2000 | SP2 |
| COMMANDER | W9260-BK03/AC NAVIGATOR//AC PATHFINDER | 2001 | SP1 |
| DT526 | D91546/D91507 | 2001 | SP3 |
| DT527 | ND74112/WASCANA//DT354 | 2001 | SP3 |
| DT528 | MEDORA/VIC | 2001 | SP3 |
| DT529 | D92288/D91430 | 2001 | SP3 |
| DT530 | D91430/D91630 | 2001 | SP3 |
| DT713 | AC AVONLEA/DT665 | 2001 | SP2 |
| DT714 | DT618/GREEN_27 | 2001 | SP1 |
| DT715 | WESTBRED 881/W9260-BK03 | 2001 | SP1 |
| DT716 | DT663/ 9469 | 2001 | SP4 |
| DT717 | DT663/ 9469 | 2001 | SP4 |
| DT718 | 92CA#93/// 4B1149/SCEPTRE//DT637 | 2001 | SP3 |
| DT719 | 8869-AJ4B/W9260-BK03 | 2001 | SP1 |
| DT720 | DT666/DT665 | 2001 | SP4 |
| DT721 | DT665/DT488 | 2001 | SP4 |
| DT534 | DT486///DT477//KAMILAROI/KYLE | 2002 | SP3 |
| DT724 | DT666/DT665 | 2002 | SP4 |
| DT726 | DT677*2/DUREX//DT662*2/GREEN_27 | 2002 | SP1 |
| DT727 | W9262-260D3/DT488 | 2002 | SP4 |
| DT728 | W9262-260D3/DT488 | 2002 | SP4 |
| DT731 | DT668/DT665 | 2002 | SP4 |
| DT732 | DT663/DT677//DT665/AC NAVIGATOR | 2002 | SP4 |
| DT733 | DT663/DT677//DT665/AC NAVIGATOR | 2002 | SP4 |
| DT734 | DT696/AC AVONLEA | 2002 | SP1 |
| DT735 | DT696/AC AVONLEA | 2002 | SP1 |
| KRONOS | KRONOS | 2002 | SP4 |
| DT535 | DT665/DT656 | 2003 | SP3 |
| DT536 | D94350/D93108 | 2003 | SP3 |
| DT537 | D94350/D93108 | 2003 | SP3 |
| DT736 | DT675/DT665//DT662 | 2003 | SP1 |
| DT737 | AC AVONLEA/DT665//AC MORSE | 2003 | SP3 |
| DT741 | DT667/DT665 | 2003 | SP4 |
| DT742 | DT667/DT665 | 2003 | SP4 |
| DT743 | WAKOOMA/AC NAVIGATOR//DT665 | 2003 | SP4 |
| DT749 | AC AVONLEA/NAPOLEON | 2003 | SP3 |
| CDC VERONA | D95253/D95212 | 2004 | SP3 |
| DT541 | DT658/DT659 | 2004 | SP3 |
| DT750 | GREEN_34/AC NAVIGATOR//DT665 | 2004 | SP4 |
| DT751 | AC MELITA/DT662//DT677 | 2004 | SP3 |
| DT756 | AC AVONLEA/ACUATICO_1//DT696 | 2004 | SP1 |
| DT757 | AC AVONLEA/ 9479-BK4 | 2004 | SP2 |
| DT760 | 920334/DT675// 9469-FU5 | 2004 | SP4 |
| DT763 | DT513/DT696 | 2004 | SP1 |
| DT546 | DT514/D97300 | 2005 | SP3 |
| DT767 | 920334/DT675// 9469-FU5 | 2005 | SP4 |
| DT768 | DT921/ 9475-CX4//DT696 | 2005 | SP1 |
| DT769 | DT921/ 9475-CX4//DT696 | 2005 | SP1 |
| DT770 | DT696/D941515 | 2005 | SP1 |
| DT771 | DT513/DT696 | 2005 | SP1 |
| DT772 | DT513/DT696 | 2005 | SP1 |
| BRIGADE | DT513/DT696 | 2005 | SP1 |
| DT774 | 9489B-EK3/DT696 | 2005 | SP1 |
| DT775 | AC PATHFINDER/DT696 | 2005 | SP1 |
| DT777 | DT691/MONGIBELLO//STRONGFIELD | 2005 | SP2 |
| DT778 | DT719/STRONGFIELD | 2005 | SP2 |
| DT779 | DT719/G9574-AZ3E | 2005 | SP1 |
| DT780 | KYLE//9560A-138/94B27-BR1C/3/DT494/4/AC NAVIGATOR/5/STRONGFIELD | 2005 | SP2 |
| DT781 | KYLE//9560A-138/94B27-BR1C/3/DT494/4/AC NAVIGATOR/5/STRONGFIELD | 2005 | SP2 |
| DT783 | 9561-AJ3A/A9800A-014//STRONGFIELD | 2005 | SP2 |
| EUROSTAR | G9575B-AA09C/DT498//DT691 | 2005 | SP1 |
| DT548 | D97437/D98074 | 2006 | SP3 |
| DT550 | D98115/AC NAVIGATOR//DT711 | 2006 | SP1 |
| DT551 | D98115/AC NAVIGATOR//DT711 | 2006 | SP1 |
| DT552 | 9469-EG2/DT711 | 2006 | SP1 |
| DT786 | DT716/STRONGFIELD | 2006 | SP2 |
| DT788 | DT719/STRONGFIELD | 2006 | SP2 |
| DT789 | DT719/STRONGFIELD | 2006 | SP2 |
| DT790 | DT719/STRONGFIELD | 2006 | SP2 |
| DT791 | DT719/STRONGFIELD | 2006 | SP2 |
| DT792 | DT719/G9574-AZ3E | 2006 | SP1 |
| DT794 | KYLE//9560A-138/94B27-BR1C/3/DT494/4/AC NAVIGATOR/5/ 9687-CA4 | 2006 | SP3 |
| DT795 | 94D11-K*3B/9468-CL5// 9688A-245D2 | 2006 | SP4 |
| DT796 | 94D11-K*3B/9468-CL5// 9688A-245D2 | 2006 | SP4 |
| DT798 | DT719/DHTON 1//STRONGFIELD | 2006 | SP2 |
| ENTERPRISE_DT787 | DT716/STRONGFIELD | 2006 | SP2 |
| DT555 | D24-1773/DT705 | 2007 | SP4 |
| DT799 | AC PATHFINDER/DT696 | 2007 | SP1 |
| DT800 | 9685-AF1A/DT721 | 2007 | SP4 |
| DT801 | DT707/DT696 | 2007 | SP1 |
| DT802 | DT707/DT696 | 2007 | SP1 |
| DT803 | 9661-AF1D/ 9586-CL5A//STRONGFIELD | 2007 | SP2 |
| DT804 | 9667A-AV6/DT704//STRONGFIELD | 2007 | SP2 |
| DT805 | 9667A-AV6/DT704//STRONGFIELD | 2007 | SP2 |
| DT806 | 9667A-AV6/DT704//STRONGFIELD | 2007 | SP2 |
| DT809 | CD98578-F-1Y-040M-040YRC-8M-1Y-0B/STRONGFIELD//STRONGFIELD | 2007 | SP2 |
| DT810 | DT721/COMMANDER//DT720 | 2007 | SP4 |
| DT811 | A9843-BE3D/DT733 | 2007 | SP4 |
| DT812 | DT721/COMMANDER//DT720 | 2007 | SP4 |
| DT557 | D99254/ 9469-EG2 | 2008 | SP4 |
| DT558 | D46-649/D76-1057 | 2008 | SP4 |
| DT559 | D99031/ 9682-AP1//DT713 | 2008 | SP2 |
| DT560 | D99031/ 9682-AP1//DT713 | 2008 | SP2 |
| DT561 | D40-2237/STRONGFIELD | 2008 | SP2 |
| DT562 | DT529/ 9468-DQ*3//STRONGFIELD | 2008 | SP2 |
| DT813 | 9667B-AA4/DT704//STRONGFIELD | 2008 | SP2 |
| DT814 | DT721/COMMANDER//DT720 | 2008 | SP4 |
| DT815 | DT721/COMMANDER//DT720 | 2008 | SP4 |
| DT816 | A0200H-082/DT735//DT733 | 2008 | SP4 |
| DT817 | DT714/DT720//DT732 | 2008 | SP4 |
| DT818 | 9675-AP2/DT732//STRONGFIELD | 2008 | SP2 |
| DT819 | COMMANDER/G9574-AZ3E//DT745 | 2008 | SP1 |
| DT820 | COMMANDER/G9574-AZ3E//DT745 | 2008 | SP1 |
| DT821 | DT739/STRONGFIELD | 2008 | SP2 |
| DT822 | DT739/STRONGFIELD | 2008 | SP2 |
| DT823 | DT744/A0100L-007//STRONGFIELD | 2008 | SP2 |
| DT824 | DT732/STRONGFIELD | 2008 | SP2 |
| DT825 | DT756/DT737 | 2008 | SP1 |
| DT565 | DT707/D84-1194 | 2009 | SP2 |
| DT831 | STRONGFIELD/DT745 | 2009 | SP2 |
| DT832 | DT749/DT735//STRONGFIELD | 2009 | SP2 |
| DT833 | A0039&DB764D18/STRONGFIELD | 2009 | SP2 |
| DT834 | DT757/STRONGFIELD | 2009 | SP2 |
| DT568 | CDC VERONA/STRONGFIELD | 2010 | SP2 |
| DT569 | DT526/DT713//STRONGFIELD | 2010 | SP2 |
| DT570 | CDC VERONA/DT732 | 2010 | SP4 |
| DT837 | A9821-JL5/DT732//DT726 | 2010 | SP4 |
| DT838 | A9821-JL5/DT732//DT726 | 2010 | SP4 |
| DT839 | DT749/STRONGFIELD | 2010 | SP2 |
| DT840 | A9918-LX2B/STRONGFIELD | 2010 | SP2 |
| DT841 | A0039&DB764D18/STRONGFIELD | 2010 | SP2 |
| DT844 | SACHEM/STRONGFIELD//DT757 | 2010 | SP2 |
| DT845 | DT769/DT751 | 2010 | SP1 |
| DT574 | STRONGFIELD/DT780 | 2011 | SP2 |
| DT575 | STRONGFIELD/DT780 | 2011 | SP2 |
| DT848 | DT769/DT751 | 2011 | SP2 |
| DT850 | A0038-360H03D/DT794 | 2011 | SP2 |
| DT851 | DT770/STRONGFIELD//A0038-360H03D | 2011 | SP2 |

^*^ Sup-population as revealed by the discriminant analysis of principal components.
