# Supplementary material for: Haplotype Loci Under Selection in Canadian Durum Wheat Germplasm Over 60 Years of Breeding: Association With Grain Yield, Quality Traits, Protein Loss, and Plant Height
Source: Front Plant Sci. 2018 Nov 5;9:1589. doi: 10.3389/fpls.2018.01589 (PMC6230583; doi:10.3389/fpls.2018.01589)
Supplement: TABLE S2 — Haplotype loci under selection in the Durum wheat Cooperative Test. [file Table_2.DOCX]

**Supplemental Table 2.** Haplotype loci under selection in the Durum wheat Cooperative test.

| Haplotypes | Markers^*^ |
| --- | --- |
| *hap_1A_1* | Tdurum_contig44888_837 |
|  | RAC875_rep_c110533_92 |
|  | BS00033749_51 |
|  | Excalibur_c9509_1180 |
| *hap_1A_2* | BS00081680_51 |
|  | Tdurum_contig83113_134 |
|  | Tdurum_contig75476_721 |
|  | IAAV6899 |
|  | Excalibur_c20777_315 |
|  | Kukri_c18434_200 |
|  | Tdurum_contig75476_167 |
| *hap_1A_3* | **Tdurum_contig50221_210** |
|  | **IAAV4099** |
|  | Kukri_c22434_513 |
|  | Ra_c2895_591 |
| *hap_1A_4* | wsnp_Ex_c41553_48351921 |
|  | Kukri_c51781_199 |
|  | IAAV5567 |
|  | Excalibur_c88792_93 |
|  | RFL_Contig3683_1784 |
| *hap_1A_5* | **RAC875_c11016_495** |
| *hap_1A_6* | BS00096930_51 |
|  | RAC875_c85550_52 |
| *hap_1B_1* | tplb0025b13_2792 |
|  | **Tdurum_contig8791_415** |
|  | RAC875_c3692_2287 |
| *hap_1B_2* | **Tdurum_contig41904_446** |
| *hap_1B_3* | BS00050126_51 |
|  | BS00062770_51 |
|  | BS00094759_51 |
|  | **BobWhite_c7333_76** |
|  | BS00047700_51 |
|  | **Excalibur_c20228_135** |
|  | **RFL_Contig1354_663** |
|  | BobWhite_c1456_615 |
|  | **BS00018461_51** |
|  | BS00073603_51 |
|  | Kukri_c30982_1173 |
|  | IACX1008 |
|  | RAC875_c50514_88 |
| *hap_1B_4* | CAP7_c3456_113 |
|  | Kukri_c667_1073 |
|  | Tdurum_contig28316_228 |
|  | Tdurum_contig56873_1237 |
|  | Tdurum_contig98080_171 |
|  | IACX3408 |
|  | Tdurum_contig44232_132 |
| *hap_1B_5* | **BS00062880_51** |
|  | **BS00075248_51** |
|  | **wsnp_Ex_c21198_30327016** |
|  | **RAC875_c1188_531** |
|  | **IACX6346** |
|  | **BobWhite_c17644_456** |
|  | **Tdurum_contig85180_99** |
| *hap_1B_6* | BobWhite_c42716_71 |
|  | **BobWhite_c8016_301** |
|  | Excalibur_rep_c96924_118 |
|  | Excalibur_c581_947 |
| *hap_1B_7* | **BS00065257_51** |
|  | **BS00087544_51** |
|  | RAC875_c26801_129 |
|  | TA004946.0577 |
|  | RAC875_c13258_955 |
|  | **RAC875_c30786_411** |
|  | **BS00089790_51** |
| *hap_1B_8* | **BS00070285_51** |
|  | **BS00099794_51** |
| *hap_1B_9* | CAP7_c1788_66 |
|  | IAAV6011 |
| *hap_1B_10* | Tdurum_contig13879_352 |
|  | Ku_c11537_539 |
|  | wsnp_Ex_c955_1827567 |
|  | BobWhite_c7691_195 |
|  | BS00001128_51 |
|  | Tdurum_contig58710_411 |
|  | Tdurum_contig92835_177 |
|  | Tdurum_contig9144_222 |
| *hap_2A_1* | **BS00097263_51** |
|  | **Ex_c4639_2851** |
|  | Excalibur_c56144_207 |
|  | **GENE.0940_83** |
|  | **RAC875_c76620_361** |
|  | **Tdurum_contig6356_150** |
|  | **CAP7_c239_267** |
|  | **Excalibur_c18267_118** |
|  | **CAP7_c11156_108** |
|  | **Excalibur_rep_c66270_789** |
|  | **wsnp_Ex_rep_c66615_64916512** |
| *hap_2A_2* | BS00055514_51 |
|  | BS00062756_51 |
| *hap_2A_3* | Excalibur_c42110_66 |
|  | Ra_c3750_270 |
|  | BobWhite_c17783_174 |
|  | BS00023144_51 |
|  | BS00030165_51 |
|  | Excalibur_c8009_325 |
|  | Kukri_rep_c110061_249 |
|  | Kukri_rep_c68300_216 |
|  | Tdurum_contig49145_914 |
|  | BS00022409_51 |
|  | IACX6200 |
|  | Tdurum_contig84245_154 |
|  | BS00062732_51 |
|  | IACX3245 |
|  | RAC875_c4609_1756 |
| *hap_2B_1* | Kukri_c16758_443 |
|  | Excalibur_c841_609 |
|  | Tdurum_contig51145_187 |
|  | Tdurum_contig51145_476 |
|  | wsnp_Ex_c18354_27181086 |
| *hap_2B_2* | BS00010318_51 |
|  | BS00070900_51 |
| *hap_2B_3* | Tdurum_contig4899_1075 |
|  | **RAC875_c34516_70** |
| *hap_2B_4* | BobWhite_c7786_376 |
|  | BS00078506_51 |
| *hap_2B_5* | BS00012071_51 |
|  | BS00070861_51 |
|  | Tdurum_contig9071_215 |
| *hap_2B_6* | BS00046164_51 |
|  | BS00046165_51 |
|  | JD_c3211_962 |
|  | BS00068310_51 |
|  | BS00064740_51 |
| *hap_2B_7* | Excalibur_c17745_493 |
|  | **BS00009060_51** |
|  | BS00043338_51 |
|  | RAC875_c35399_497 |
| *hap_2B_8* | **BobWhite_c7274_333** |
| *hap_2B_9* | **BobWhite_c20158_834** |
|  | **BS00025106_51** |
|  | IAAV8570 |
| *hap_3A_1* | BS00065956_51 |
|  | Tdurum_contig60260_89 |
|  | Tdurum_contig99640_243 |
|  | Tdurum_contig91865_242 |
| *hap_3A_2* | BS00065468_51 |
| *hap_3A_3* | Tdurum_contig93364_355 |
| *hap_3A_4* | Tdurum_contig55335_316 |
|  | Tdurum_contig56748_632 |
| *hap_3A_5* | **Ra_c11263_2353** |
|  | **Tdurum_contig68855_91** |
|  | **BobWhite_c34548_96** |
|  | **BS00026189_51** |
|  | BS00031289_51 |
|  | **CAP7_c3367_68** |
|  | **Kukri_rep_c70479_411** |
|  | RAC875_c744_1935 |
|  | **TA002508.0190** |
|  | **wsnp_Ex_c45213_51068305** |
|  | Tdurum_contig11121_739 |
|  | BS00070511_51 |
|  | Ku_c68484_1276 |
|  | Tdurum_contig70196_106 |
|  | wsnp_Ex_c37208_45002588 |
|  | wsnp_Ra_c35889_44345459 |
|  | BS00101401_51 |
|  | BS00003971_51 |
|  | wsnp_Ex_c28930_38008757 |
|  | IAAV902 |
|  | Kukri_rep_c87640_135 |
|  | IACX787 |
|  | Tdurum_contig11714_304 |
| *hap_3A_6* | IACX333 |
|  | BobWhite_c38444_238 |
|  | BS00023337_51 |
| *hap_3B_1* | BS00064312_51 |
|  | Tdurum_contig60949_306 |
|  | Kukri_c73725_218 |
|  | **CAP8_c1799_237** |
| *hap_3B_2* | **Tdurum_contig12643_514** |
|  | **Tdurum_contig93431_485** |
|  | Tdurum_contig48528_144 |
|  | Tdurum_contig48528_320 |
|  | Tdurum_contig67350_494 |
| *hap_3B_3* | Tdurum_contig60330_65 |
| *hap_4A_1* | Kukri_c8815_531 |
| *hap_4A_2* | BobWhite_rep_c63429_271 |
|  | wsnp_Ex_rep_c104859_89444355 |
| *hap_4A_3* | BS00030838_51 |
| *hap_4A_4* | **Excalibur_c4325_1440** |
| *hap_4A_5* | wsnp_Ra_c22775_32274079 |
|  | **Ra_c60252_1733** |
|  | **Ra_c60252_743** |
|  | Ra_c1897_2401 |
|  | Tdurum_contig43961_607 |
|  | **BS00091561_51** |
|  | **BS00110021_51** |
|  | GENE.4739_457 |
|  | GENE.4750_495 |
|  | Tdurum_contig44771_1945 |
|  | Tdurum_contig44771_984 |
|  | **Tdurum_contig93100_640** |
|  | RFL_Contig3841_2409 |
|  | RFL_Contig3841_2433 |
|  | Tdurum_contig75819_1309 |
|  | Tdurum_contig75819_1591 |
|  | BS00039811_51 |
|  | Tdurum_contig10482_110 |
|  | BS00010006_51 |
|  | **Tdurum_contig51959_1408** |
|  | **BS00104640_51** |
|  | **tplb0032m13_1358** |
|  | **Excalibur_rep_c112003_621** |
|  | Tdurum_contig44771_931 |
|  | **Tdurum_contig10654_375** |
|  | **Tdurum_contig27944_266** |
|  | **Tdurum_contig47476_441** |
|  | **RAC875_c82470_174** |
| *hap_4A_6* | D_F1BEJMU01CBKJF_321 |
|  | Tdurum_contig14123_802 |
|  | RAC875_c55173_65 |
| *hap_4B_1* | RAC875_c12959_869 |
|  | IAAV2271 |
|  | **BobWhite_c30050_125** |
|  | **Tdurum_contig74720_539** |
|  | **Tdurum_contig68677_480** |
|  | Tdurum_contig55414_154 |
|  | Tdurum_contig41902_730 |
| *hap_4B_2* | Tdurum_contig69405_332 |
|  | Tdurum_contig57212_71 |
| *hap_4B_3* | Kukri_c7241_322 |
| *hap_4B_4* | Tdurum_contig29247_404 |
|  | Tdurum_contig9893_492 |
|  | Tdurum_contig9893_571 |
|  | BS00022808_51 |
|  | Excalibur_rep_c114140_567 |
|  | wsnp_Ex_c21293_30421496 |
|  | BS00040517_51 |
| *hap_5A_1* | BS00021660_51 |
|  | BS00046772_51 |
| *hap_5A_2* | Tdurum_contig10398_2166 |
|  | Tdurum_contig61258_264 |
| *hap_5A_3* | tplb0039m09_92 |
|  | GENE.3189_377 |
|  | IAAV9057 |
|  | RAC875_c4033_192 |
|  | RAC875_c95309_460 |
|  | wsnp_Ex_c43578_49857984 |
|  | BS00067453_51 |
|  | BS00089967_51 |
|  | Tdurum_contig43413_1432 |
|  | Tdurum_contig50282_211 |
| *hap_5A_4* | BS00068178_51 |
|  | wsnp_Ex_c18941_27840714 |
| *hap_5A_5* | BS00074299_51 |
|  | Tdurum_contig86202_175 |
|  | BS00074301_51 |
|  | Tdurum_contig86202_145 |
|  | **Excalibur_c29304_176** |
| *hap_5B_1* | BS00063476_51 |
|  | RFL_Contig1899_2863 |
|  | wsnp_Ku_rep_c103274_90057407 |
|  | Tdurum_contig53796_360 |
|  | Tdurum_contig9291_438 |
|  | Tdurum_contig9291_744 |
| *hap_5B_2* | wsnp_Ku_c3102_5811860 |
|  | Kukri_c35352_483 |
|  | RAC875_c36779_148 |
|  | BS00087043_51 |
|  | CAP12_c703_150 |
|  | Kukri_c37692_347 |
|  | RAC875_c10139_92 |
|  | BS00020982_51 |
|  | Excalibur_c33675_410 |
|  | **Tdurum_contig82805_271** |
|  | BS00068805_51 |
|  | BS00068711_51 |
|  | Tdurum_contig10268_1000 |
|  | Tdurum_contig16866_148 |
|  | **Tdurum_contig28016_254** |
| *hap_5B_3* | **BS00022065_51** |
|  | BobWhite_rep_c60245_107 |
|  | **Tdurum_contig43078_437** |
|  | BS00065029_51 |
|  | Kukri_c45951_367 |
| *hap_5B_4* | **Kukri_c16864_398** |
| *hap_5B_5* | RAC875_c20161_821 |
| *hap_5B_6* | Excalibur_c2207_1060 |
|  | wsnp_Ku_c16116_24914991 |
| *hap_6A_1* | BobWhite_c5092_422 |
|  | BS00105910_51 |
|  | BS00105913_51 |
|  | Ex_c2978_643 |
|  | Excalibur_c4202_1326 |
|  | GENE.1530_1075 |
|  | RAC875_rep_c97032_73 |
|  | TA001668.0792 |
| *hap_6A_2* | BobWhite_c10740_179 |
|  | Excalibur_c60006_452 |
|  | RAC875_c47243_145 |
|  | Tdurum_contig42906_732 |
|  | Tdurum_contig62141_93 |
|  | Tdurum_contig64407_187 |
|  | Tdurum_contig9544_2126 |
|  | Tdurum_contig4964_569 |
| *hap_6A_3* | Excalibur_c48569_78 |
|  | Tdurum_contig55124_310 |
|  | Kukri_c22149_276 |
|  | Tdurum_contig16290_156 |
| *hap_6A_4* | Ra_c29420_237 |
|  | Tdurum_contig59599_1213 |
|  | Tdurum_contig75737_1479 |
|  | Tdurum_contig83933_258 |
| *hap_6B_1* | wsnp_Ku_c2119_4098330 |
|  | RAC875_rep_c77197_187 |
| *hap_6B_2* | BS00109708_51 |
|  | Tdurum_contig92921_193 |
|  | Ex_c61830_971 |
|  | Kukri_c24439_323 |
| *hap_6B_3* | **wsnp_CAP11_rep_c4300_2030142** |
|  | **Tdurum_contig49982_372** |
|  | **Tdurum_contig28405_288** |
|  | **BobWhite_c10614_157** |
|  | **RAC875_c47717_388** |
|  | Tdurum_contig48689_514 |
|  | **wsnp_Ra_c2730_5190365** |
|  | BS00028217_51 |
|  | RAC875_rep_c104354_333 |
|  | Tdurum_contig45714_427 |
|  | **wsnp_Ex_c17435_26144201** |
| *hap_6B_4* | wsnp_Ku_c1876_3666308 |
| *hap_6B_5* | **Kukri_c65766_78** |
|  | IAAV951 |
| *hap_7A_1* | Tdurum_contig955_254 |
| *hap_7A_2* | Tdurum_contig85217_206 |
|  | Tdurum_contig85217_286 |
|  | BS00069242_51 |
| *hap_7A_3* | Tdurum_contig82438_73 |
|  | **CAP7_c548_299** |
|  | Ex_c499_767 |
|  | Excalibur_rep_c81365_214 |
|  | **IAAV1940** |
|  | **Tdurum_contig59789_174** |
|  | **Tdurum_contig67992_160** |
|  | Tdurum_contig28195_198 |
|  | **Tdurum_contig8190_280** |
|  | Tdurum_contig12263_179 |
|  | Tdurum_contig31137_373 |
|  | **BS00021692_51** |
|  | **BS00102772_51** |
|  | **BS00102773_51** |
|  | **Tdurum_contig85377_141** |
|  | **Tdurum_contig42712_284** |
|  | Tdurum_contig51645_738 |
| *hap_7A_4* | **BS00040657_51** |
|  | Excalibur_c987_197 |
|  | **wsnp_JG_c227_167774** |
| *hap_7A_5* | **Tdurum_contig42661_202** |
| *hap_7A_6* | wsnp_BE498209A_Ta_2_1 |
| *hap_7A_7* | BS00067759_51 |
|  | IAAV4430 |
|  | Kukri_rep_c85030_287 |
|  | Ra_c8425_1331 |
|  | Tdurum_contig11862_341 |
|  | wsnp_be471272A_Ta_2_1 |
|  | Tdurum_contig76441_240 |
|  | BS00063933_51 |
|  | BS00077560_51 |
|  | Kukri_rep_c68087_688 |
|  | BS00063383_51 |
|  | BS00065647_51 |
|  | BS00079590_51 |
|  | RAC875_c37071_73 |
|  | RAC875_c37565_396 |
|  | Tdurum_contig28843_80 |
|  | wsnp_Ex_rep_c71217_70021470 |
|  | wsnp_Ra_c23253_32762188 |
|  | Ex_c21222_313 |
|  | Ra_c3267_787 |
|  | Tdurum_contig11542_548 |
|  | Tdurum_contig62237_531 |
|  | Tdurum_contig70105_162 |
|  | wsnp_CAP11_c592_400447 |
|  | Ra_c66215_1118 |
|  | BobWhite_c24259_117 |
|  | wsnp_Ex_rep_c105174_89674370 |
|  | wsnp_Ex_c45495_51274052 |
| *hap_7A_8* | wsnp_Ex_c7001_12062578 |
|  | IAAV8081 |
|  | RAC875_c13696_226 |
|  | wsnp_CAP11_c827_513472 |
| *hap_7A_9* | RAC875_c43295_135 |
|  | Tdurum_contig69067_405 |
|  | BS00067425_51 |
|  | BS00067610_51 |
|  | BS00071478_51 |
|  | BS00088825_51 |
|  | BS00094965_51 |
|  | RAC875_c37085_317 |
| *hap_7A_10* | IAAV5578 |
| *hap_7A_11* | Tdurum_contig59633_56 |
|  | Tdurum_contig93328_869 |
|  | BS00002510_51 |
| *hap_7B_1* | Tdurum_contig9465_925 |
| *hap_7B_2* | **Tdurum_contig43523_359** |
|  | Tdurum_contig49572_643 |
|  | Tdurum_contig51024_1234 |
|  | **Ku_c665_985** |
| *hap_7B_3* | BS00075332_51 |
|  | Tdurum_contig41998_1213 |
|  | CAP7_rep_c5216_143 |
|  | Ku_c10179_1837 |
| *hap_7B_4* | RAC875_c3361_180 |
|  | wsnp_Ex_c10571_17258682 |
| *hap_7B_5* | Tdurum_contig43435_417 |
| *hap_7B_6* | Excalibur_c2062_383 |

^*^ Markers that showed complete reversal of allelic state are highlighted in bold
