# Supplementary material for: Haplotype Loci Under Selection in Canadian Durum Wheat Germplasm Over 60 Years of Breeding: Association With Grain Yield, Quality Traits, Protein Loss, and Plant Height
Source: Front Plant Sci. 2018 Nov 5;9:1589. doi: 10.3389/fpls.2018.01589 (PMC6230583; doi:10.3389/fpls.2018.01589)
Supplement: TABLE S3 — Haplotype loci associated with traits and containing SNPs with complete reversal of allelic state. [file Table_3.DOCX]

**Supplemental Table 3.** Haplotype loci associated with traits and containing SNPs with complete reversal of allelic state.

| Haplotypes | Traits | nb_R^1^ | Nb_SNPs^2^ |
| --- | --- | --- | --- |
| *hap_1A_3* | Semolina pigment | 2 | 4 |
| *hap_1B_2* | Gluten index, Dough tenacity, Dough extensibility, Deformation energy | 1 | 1 |
| *hap_1B_3* | Semolina pigment | 4 | 13 |
| *hap_1B_5* | Semolina pigment | 7 | 7 |
| *hap_1B_6* | Semolina pigment | 1 | 4 |
| *hap_1B_7* | Protein content, Semolina pigment | 4 | 7 |
| *hap_1B_8* | Grain yield | 2 | 2 |
| *hap_2A_1* | Plant height, Dough extensibility | 10 | 11 |
| *hap_2B_7* | Gluten index, Semolina pigment, Grain yield | 1 | 4 |
| *hap_2B_9* | Protein content, Protein loss, Deformation energy | 2 | 3 |
| *hap_3A_5* | Semolina pigment | 8 | 23 |
| *hap_3B_2* | Semolina pigment | 2 | 5 |
| *hap_4A_4* | Dough tenacity, Dough extensibility | 1 | 1 |
| *hap_4B_1* | Plant height, Pasta b*, Dough extensibility, Pigment loss, Grain yield | 3 | 7 |
| *hap_5A_5* | Semolina pigment | 1 | 5 |
| *hap_5B_4* | Semolina pigment | 1 | 1 |
| *hap_6B_3* | Semolina pigment | 7 | 11 |
| *hap_6B_5* | Dough tenacity | 1 | 2 |
| *hap_7A_3* | Semolina pigment | 10 | 17 |
| *hap_7A_4* | Semolina pigment | 2 | 3 |
| *hap_7A_5* | Semolina pigment | 2 | 2 |

^1^ Number of SNPs that showed a complete reversal of allelic.

^2^ Total number of SNPs.
